# Genome-Wide Discovery of miRNAs with Differential Expression Patterns in Responses to Salinity in the Two Contrasting Wheat Cultivars

**DOI:** 10.3390/ijms222212556

**Published:** 2021-11-21

**Authors:** Muhammad Zeeshan, Cheng-Wei Qiu, Shama Naz, Fangbin Cao, Feibo Wu

**Affiliations:** 1 Department of Agronomy, College of Agriculture and Biotechnology, Zijingang Campus, Zhejiang University, Hangzhou 310058, China; 11616102@zju.edu.cn (M.Z.); 11716027@zju.edu.cn (C.-W.Q.); shamaktk@yahoo.com (S.N.); caofangbin@zju.edu.cn (F.C.); 2 Key Laboratory of Crop Cultivation and Tillage, Agricultural College of Guangxi University, Nanning 530004, China; 3 Jiangsu Co-Innovation Center for Modern Production Technology of Grain Crops, Yangzhou University, Yangzhou 225009, China

**Keywords:** miRNAs, high-throughput sequencing, roots, wheat, salt stress

## Abstract

Salinity is a serious environmental issue. It has a substantial effect on crop yield, as many crop species are sensitive to salinity due to climate change, and it impact is continuing to increase. Plant microRNAs (miRNAs) contribute to salinity stress response in bread wheat. However, the underlying molecular mechanisms by which miRNAs confer salt tolerance in wheat are unclear. We conducted a genome-wide discovery study using Illumina high throughput sequencing and comprehensive in silico analysis to obtain insight into the underlying mechanisms by which small RNAs confer tolerance to salinity in roots of two contrasting wheat cvv., namely Suntop (salt-tolerant) and Sunmate (salt-sensitive). A total of 191 microRNAs were identified in both cultivars, consisting of 110 known miRNAs and 81 novel miRNAs; 181 miRNAs were shared between the two cultivars. The known miRNAs belonged to 35 families consisted of 23 conserved and 12 unique families. Salinity stress induced 43 and 75 miRNAs in Suntop and Sunmate, respectively. Among them, 14 and 29 known and novel miRNAs were expressed in Suntop and 37 and 38 in Sunmate. In silico analysis revealed 861 putative target mRNAs for the 75 known miRNAs and 52 putative target mRNAs for the 15 candidate novel miRNAs. Furthermore, seven miRNAs including tae-miR156, tae-miR160, tae-miR171a-b, tae-miR319, tae-miR159a-b, tae-miR9657 and novel-mir59 that regulate auxin responsive-factor, SPL, SCL6, PCF5, R2R3 MYB, and CBL-CIPK, respectively, were predicted to contribute to salt tolerance in Suntop. This information helps further our understanding of how the molecular mechanisms of salt tolerance are mediated by miRNAs and may facilitate the genetic improvement of wheat cultivars.

## 1. Introduction

Non-coding RNAs are functional RNAs with low protein-coding potential and are divided into three main groups based on length: small (18–30 nt), medium (31–200 nt), and long (>201 nt) [1]. Specifically, small RNAs (sRNAs) are 21–24 nt long, are predominantly derive from intergenic region [2], and are vital regulators of protein-coding transcript expression at both RNA and DNA levels. These regulatory sRNAs mainly function in two ways: by posttranscriptional gene silencing (PTGS) through mRNA degradation, mRNA cleavage, or translational repression; and by transcriptional gene silencing (TGS) due to histone modifications [3,4,5]. It is well known that plant miRNAs are responsible for the regulation of many genes involved in several important biological and metabolic pathways, such as RNA metabolism, signal transduction, plant organ development, and response to pathogens [6,7,8]. In addition, many miRNAs play crucial roles in adaptation to abiotic stresses, such as salinity [9], cold [10], drought [11], and heavy metals [12] in plant species. For example, Kuang et al. [9] found that miR159a, miR169i, miR319a/miR396e module, and miR172b were down-regulated under salinity stress in barly, by regulating their target proteins MYB33, NHX1/LEA7, TCP4, GRFs, and *Hv*AP2, respectively, suggesting that they might play a role in salt tolerance. The other evidence for a role of miRNAs in stress tolerance are the effects of *Osa*-mir319 on cold tolerance in transgenic rice seedling [13] and the over-expression of *Sly*-mir169c resulting in limited stomatal opening, lowered relative water content, decreased transpiration rates, and enhanced drought tolerance in tomato plants [14]. Approximately 40 miRNAs families have been identified as being involved in plant responses to environmental stresses, many of which are involved in responses to drought and heavy metals [15]. Some miRNAs are conserved among species while others are non-conserved, called novel miRNAs and are often tissue or species-specific [16]. Due to their involvement in stress responses, an understanding of the expression of miRNAs is important, especially for breeding crops that are tolerant of abiotic stress.

Wheat (*Triticum aestivum* L.) is the most important food crop and is grown globally in diverse environments. It is considered as the most important crop used for human diets among the cereals [17]. Worldwide, it provides about 20% of calories for human consumption, and improving its productivity will increase global food security [18]. However, being a salt-sensitive crop, wheat production and yield will be compromised in near future due to increasing soil salinity. Therefore, the breeding of salt-resistant and high yielding varieties is important for food security. This can be achieved through pre-breeding programs that transfer genes responsible for salt tolerance from related species into wheat [19]. However, the genetic regulatory networks and signaling pathways involved in salinity remain to be elucidated.

In nature, plants, as with many sessile organisms, have adopted different structural and functional responses to cope with a wide range of environmental stimuli and stresses [2,20]. Most crop plants can only tolerate minor fluctuations in their growth conditions, and any fluctuations beyond a threshold leads to stress. These stresses limit the growth and reproduction of crops [21]. In plant–environment co-evolution, a series of avoidance and/or tolerance mechanisms have evolved at the morphological, physiological, biochemical, cellular, and molecular levels to impart stress tolerance and involve a variety of signal transduction pathways. Consequently, plants can utilize multiple gene regulatory networks to adjust metabolic processes [22] and restore the cellular homeostasis [1]. Emerging studies have shown that miRNAs play a significant role in the regulation of stress-related gene expression [23]. Several miRNAs are differentially expressed under UV-B radiation, such as miR159, miR167a, and miR171, miR156, miR164, and miR395 in wheat [24]. Similarly, other miRNA families, such as miR396, miR169, and miR156, are differentially expressed under saline conditions in different species, and suggests that members of these families may alter gene expression under salinity stress in a species-specific manner [25]. Moreover, overexpression of *gma*-miR172 in *Arabidopsis* [26] and *osa*-miR156 in rice resulted in enhanced tolerance to salinity and cold stresses, respectively [13]. However, the study of wheat microRNAs and their role in tolerant of salt stress is still limited.

It is estimated that up to 1.2 million ha of land is lost to salinization each year [27], climate change is exacerbating this situation. As bread wheat is a salinity-sensitive species, it is important to gain a better understanding of the factors affecting salinity tolerance. Previously, we compared two bread wheat cultivars, Suntop and Sunmate, with the salinity-tolerant barley cultivar, CM72, and found that Suntop exhibited a high level of salinity tolerance similar to CM72 [28]. Given the tolerance of Suntop, this study was designed to profile miRNAs expression using Illumina HiSeq 2000 RNA sequencing of its roots. The results from this study provide valuable information that will help our understanding of the regulatory roles of miRNAs in the salt stress tolerance mechanism of Suntop.

## 2. Results

### 2.1. Total ATPase Activity and Total Amino Acid and Soluble Sugar Contents

**Figure 1:**
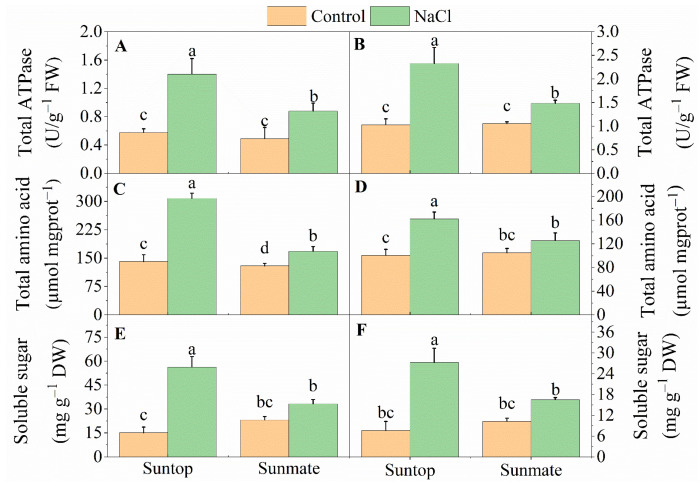
Total ATPase activity (**A**,**B**), contents of total amino acids (**C**,**D**) and soluble sugars (**E**,**F**) in leaves (left panel) and roots (right panel) of two wheat cultivars, Suntop and Sunmate, exposed to 100 mM NaCl for five days. These data are means ± SE (*n* = 4). Different letters indicate significant differences at *p*-values = 0.05.

Under saline conditions, both cultivars (Suntop and Sunmate) had increased total ATPase activities, and contents of total amino acids and soluble sugars in both their leaves and roots (Figure 1A–E). However, the increase were greater for Suntop than for Sunmate. When compared with the control, there were 2.4-, 2.1- and 3.7-fold increases in the leaves and 2.2-, 1.6- and 3.5-fold increases in the roots of Suntop for total ATPase activity, amino acids and soluble sugar contents, respectively. However, these increases were marginal in Sunmate which recorded 1.3-, 1.8-, and 1.1-fold increases in these features in the leaves and 1.2-, 1.4-, and 1.6-fold increases in the roots, respectively.

### 2.2. MicroRNAs Profiling Response to Salinity Stress

To study the sRNA transcriptome of wheat, four cDNA libraries (ST-CK, ST-T, SM-CK and SM-T) derived from the roots of normal and salinity-treated wheat plants were constructed and sequenced using high throughput sequencing (HTS). The preliminary results obtained from the four libraries are presented in Table 1. The sRNA sequencing generated 29,114,838, 28,580,752.5, 28,973,938.5 and 29,753,197 raw reads from the ST-CK, ST-T, SM-CK and SM-T libraries, respectively. After filtering the low-quality sequences, polyA/T/G/C sequences, adapters, and small sequences (smaller than16 nt long), 27,175,593.5 (95.08%), 26,292,555 (90.31%), 25,900,067 (89.39%), and 25,819,464 (86.77%) clean reads were obtained, respectively. Q20 percentage values of clean reads were more than 99% for the all libraries, indicating that results from analysis of the HTS were reliable. We used downstream analysis to align the clean tags with sRNA databases such as miRBase (http://www.mirbase.org, accessed on 18 October 2021) and Rfam in order to remove the corresponding fragments of non-coding RNAs; this resulted in 24,908,414 (St-CK) and 24,352,075.5 (ST-T) mapped reads remaining in Suntop and 24,230,503.5 (SM-CK) and 22,175,264 (SM-T) remaining in Sunmate (Table 1).

### 2.3. Identification of Known and Novel miRNAs

Candidate miRNAs were predicted following the criteria given in Jeong et al. [29]. Subsequently, unique reads that aligned to the miRNA database (miRBase 21.0) with less than two mismatches were considered known and novel miRNAs. In total, 191 candidate miRNAs were obtained in the four libraries; 181 miRNAs were co-expressed in Suntop and Sunmate and 10 miRNAs each were exclusively expressed in Suntop and Sunmate (Figure S1A). We further found that the 66 known miRNAs are members of 35 miRNA families, of which the families MIR1120 and MIR1122 contained the highest number of members (seven members). MIR9657 and MIR9666 contained 4 miRNAs each and MIR159 and MIR167_1 contained 3 members; 7 families contained 2 members and 20 families had 1 representative. However, 43 miRNAs did not belong to any known family (Table S1). As known sRNAs are 21 to 24 nts in length, we analyzed the length distribution of clean tag sRNAs of the four libraries. The majority of the sRNA had lengths ranging 18 to 30 nts, with the most abundant lengths being 19, 21, and 24 nts in the four libraries, which is consistent with the typical size range of dicer-derived miRNAs. In each treatment, the sRNAs in both cultivars showed similar patterns of length distributions. However, there was a significant difference in the relative abundance of sRNAs in each cultivar. The number of 19, 21, and 24 nts are 9.3%, 11%, and 13.6% in Suntop as compared with Sunmate in the control libraries respectively. For the libraries from NaCl-treated plants, there were 15.9, 12.2, and 13% more sRNAs that were 19, 21, and 24 nts in length in Suntop than in Sunmate (Figure S1B,C), a finding that is in agreement with microRNAs trends previously reported [30,31].

### 2.4. Evolutionary Conservation Analysis of Known miRNAs Families

To determine whether the miRNA found in bread wheat were evolutionary conserved among the plant species, we used the sequences of the miRNAs in the 35 families of the known miRNAs to see whether they could be found in the 37 other plant species in the miRNA database (http://www.mirbase.org, accessed on 27 April 2019). We divided all sequences into those from conserved families and those that were wheat-specific miRNAs based on whether they were already reported in other plants species [32]. It was found that among the 35 families, 4 families (MIR9654, MIR9657, MIR9662, and MIR9666) were unique to *Triticum aestivum* and 8 families (including MIR5084, MIR7757, MIR9672, MIR9674, MIR9677, MIR9772, MIR9776, and MIR9783) were detected in both *Triticum aestivum* and *Aegilops tauschi*. The analysis further found that two miRNA families (MIR169 and MIR408) could be found in 30 plants species and 3 miRNA families (MIR160, MIR164 and MIR398) were found in 28 plants species, suggesting a close evolutionary relationship of *Triticum aestivum* with other plants species (Table S2).

### 2.5. Identification of miRNAs Related to Salt Stress

**Figure 2:**
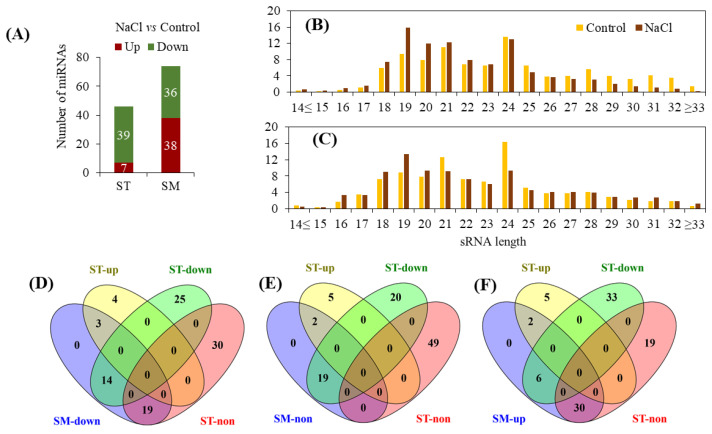
Analysis of the differentially expressed miRNAs from the roots of Suntop (ST) and Sunmate (SM). (**A**) Number of miRNAs in control and NaCl-treated roots of the two wheat cultivars. Length distributions of differentially expressed (**B**) known and (**C**) novel miRNA in the roots of both cultivars. The Venn diagrams show the number of miRNAs regulated by the salinity treatment and the overlapping patterns between the two cultivars. The data are overlaps of miRNA in Suntop, which are down-regulated (**D**), unchanged (**E**) and up-regulated (**F**) in Sunmate. Within each genotype, miRNAs having fold change (salinity vs. control) of log_2_ N ≥ 1 are considered as up-regulated, those between 0 < |log_2_ N| < 1 are considered as unchanged, and those with a change of log_2_ N ≤ −1 are considered as down-regulated, *p* < 0.01.

**Figure 3:**
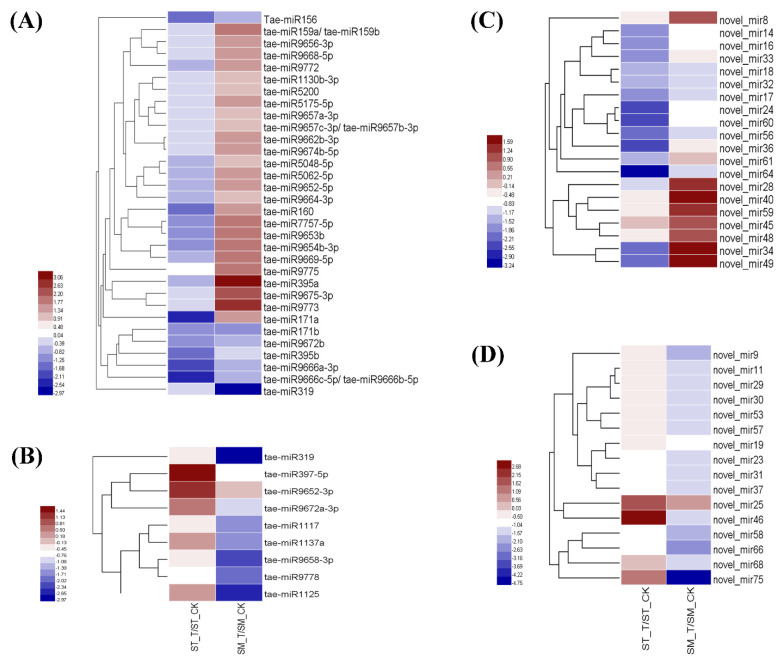
The hierarchical clustering analysis of the predicted target genes of differentially expressed conserved (**A**,**B**) and novel (**C**,**D**) miRNAs in roots between Suntop (ST) and Sunmate (SM) in response to 100 mM NaCl. (**A**) Conserved miRNAs (Table S3) in roots whose expression was unchanged in ST but up-regulated in SM, or down-regulated in ST but up-regulated or unchanged in SM. (**B**) Conserved miRNAs (Table S4) in roots whose expression was unchanged in ST but down-regulated in SM, or up-regulated in ST but down-regulated or unchanged in SM. (**C**) Novel miRNAs (Table S5) in roots whose expression was unchanged in ST but up-regulated in SM, or down-regulated in ST but up-regulated or unchanged in SM. (**D**) Novel miRNAs (Table S6) in roots whose expression was unchanged in ST but down-regulated in SM, or up-regulated in ST but down-regulated or unchanged in SM.

This study was motivated by our interest in miRNAs and explored their potential biological roles in the modulation of gene expression in wheat subjected to salt stress. From a total of 191 identified (known and novel) miRNAs in the two cultivars, 46 known miRNAs and 52 novel miRNAs were differentially expressed (DE) (|log_2_ fold-change| ≥ 1 and *p*-value ≤ 0.01) and were either up-regulated or down-regulated under the salinity treatment (Table S1). Out of those, 7 and 39 miRNAs in Suntop and 38 and 36 miRNAs in Sunmate were up-regulated and down-regulated, respectively (Figure 2A). The length distribution of these differentially expressed miRNAs showed that those consisting of 21 nts were the most abundant size class of the known miRNAs followed by those containing 22 nts (Figure 2B), while the most abundant size for the novel miRNAs were 23 and 24 nts (Figure 2C). Overall, 7, 49 and 39 miRNAs were up-regulated, unaltered or down-regulated, respectively, in response to salt stress in Suntop but in Sunmate, the corresponding numbers were 36, 21, and 38 (Figure 2D–F). Among the known miRNAs, 10 miRNAs (tae-miR156, tae-miR160, tea-miR171a, tae-miR171b, tae-miR395b, tae-miR7757-5p, tae-miR9653b, tae-miR9666a-3p, tae-miR9666c-5p-tae-miR9666b-5p, and tae-miR9672b) were down-regulated in Suntop (ST) but either unaltered or up-regulated in Sunmate (SM), similarly 21 miRNAs were unaltered in Suntop (ST) but were up-regulated in Sunmate (SM) (Table S3, Figure 3A). In addition, seven miRNAs (tae-miR319, tae-miR1117, tae-miR1125, tae-miR1137a, tae-miR9658-3p, tae-miR9672a-3p, and tae-miR9778) were down-regulated in Sunmate (SM) but unchanged in Suntop (ST); similarly, two miRNAs (tae-miR397-5p and tae-miR9652-3p) were unchanged in Sunmate but up-regulated in Suntop (Table S4, Figure 3B). Four differentially expressed known miRNAs (tae-miR398; tae-miR9662a-3p; tae-miR9666b-3p; tae-miR9774) had similar expression patterns in both cultivars (Table S1). Analysis of the 46 conserved miRNAs showed that 13 of them could not be with target genes; 20 had multiple targets, and a further 13 miRNAs had single target gene (Table S1).

**Figure 4:**
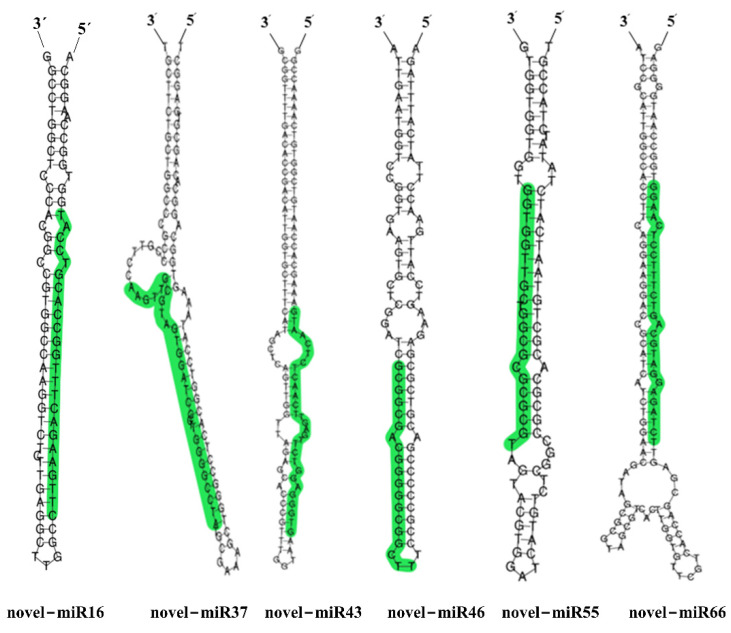
Predicted secondary structure of six novel precursor miRNAs differently expressed in Suntop and Sunmate in response to salinity stress. Green shading indicates the sequence of the mature miRNAs.

Fifty differentially expressed novel miRNAs were detected in the two cultivars (Table S1). Among them, 14 novel miRNAs were down-regulated in Suntop but either up-regulated or unchanged in Sunmate. In addition, seven novel miRNAs were unchanged in Suntop but up-regulated in Sunmate (Table S5, Figure 3C). Furthermore, 15 novel-miRNAs were down-regulated in Sunmate but unchanged in Suntop (Table S6, Figure 3D). The 50 novel miRNA could be distinguished from other sRNAs due to their precursor hairpin structure [33]. The putative secondary structures of six of the novel miRNAs (novel-miR16, novel-miR37, novel-miR43, novel-miR46, novel-miR55, and novel-miR66) are predicted in Figure 4.

### 2.6. Validation of Targets mRNA and miRNA with qRT-PCR

**Figure 5:**
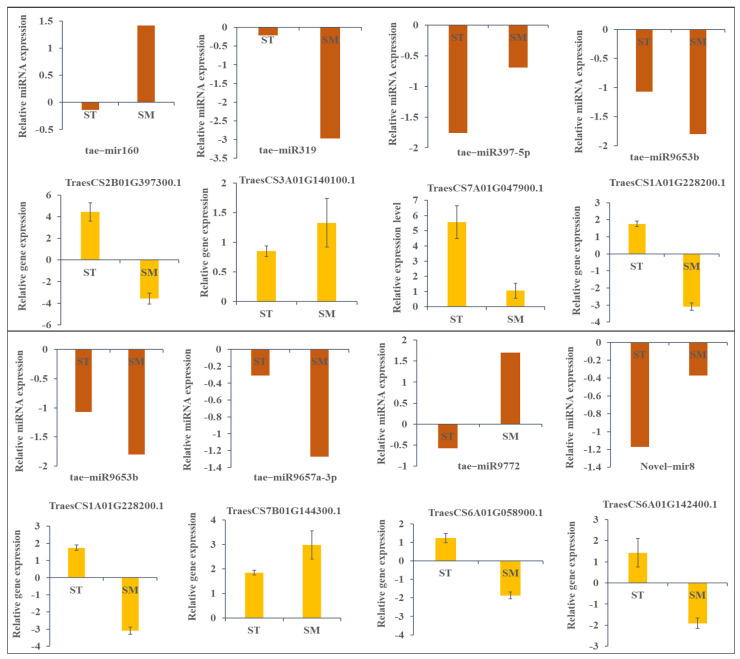
Validation of eight differentially expressed miRNAs and target genes in roots of Suntop (ST) and Sunmate (SM) from HTS analyses. Relative miRNA expression is the fold change in normalized reads from the sequencing of sRNAs between the NaCl treatment and the control. Fold change was calculated as log_2_ (NaCl reads/control reads). Data related to miRNA expression are the means of two biological replicates, while the data from the gene expression studies are the means of three biological replicates and two technical replicates.

**Figure 6:**
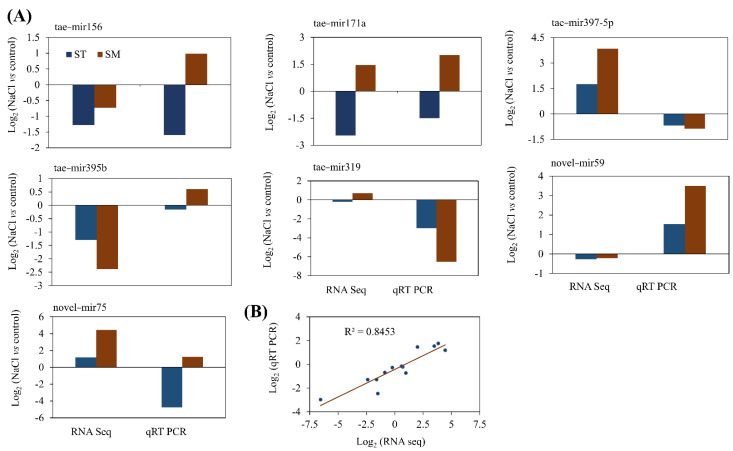
qRT-PCR confirmation of seven miRNAs identified in Suntop and Sunmate whose expression changed in response to salinity stress. (**A**) Expression patterns of seven miRNAs in the qRT-PCR and sequencing dataset. (**B**) Regression analysis of the RNA-Seq log_2_ value and real time PCR values.

We used quantitative RT-PCR to validate and quantify the expression of the miRNA targets (mRNAs) and eight target mRNAs (*Traes*CS2B01G397300.1, *Traes*CS3A01G140100.1, *Traes*CS7A01G047900.1, *Traes*CS1A01G228200.1, *Traes*CS7B01G144300.1, *Traes*CS6A01G058900.1 *Traes*CS1D01G021300.1, and *Traes*CS6A01G142400.1) were randomly selected for this process (Figure 5). Gene specific primers were used to amplify the targeted genes and to calculate the relative expression in each qPCR reaction. It should be noted that an inverse expression pattern is expected between miRNAs and their corresponding mRNA targets. Surprisingly, in Sunmate under the NaCl treatment, we found two mRNAs (TraesCS1A01G228200.1 and TraesCS6A01G142400.1) that were down-regulated like their respective miRNAs. However, the expression patterns of the other mRNA and miRNA pairs were as expected suggesting that the quality was high for the RNA-Seq datasets. Seven miRNAs (tae-miR156, tae-miR171a, tae-miR319, tae-miR395b, tae-miR397-5P, novel-miR59 and novel-miR75) were selected and their expression patterns checked using qRT-PCR (Figure 6A). Regression analysis, as shown in the Figure 6B, shows a strong, inverse relationship between the qRT-PCR results and the sequencing data (R^2^ = 0.8453).

### 2.7. Prediction of Target Genes and Their Associated Regulatory Pathways

**Figure 7:**
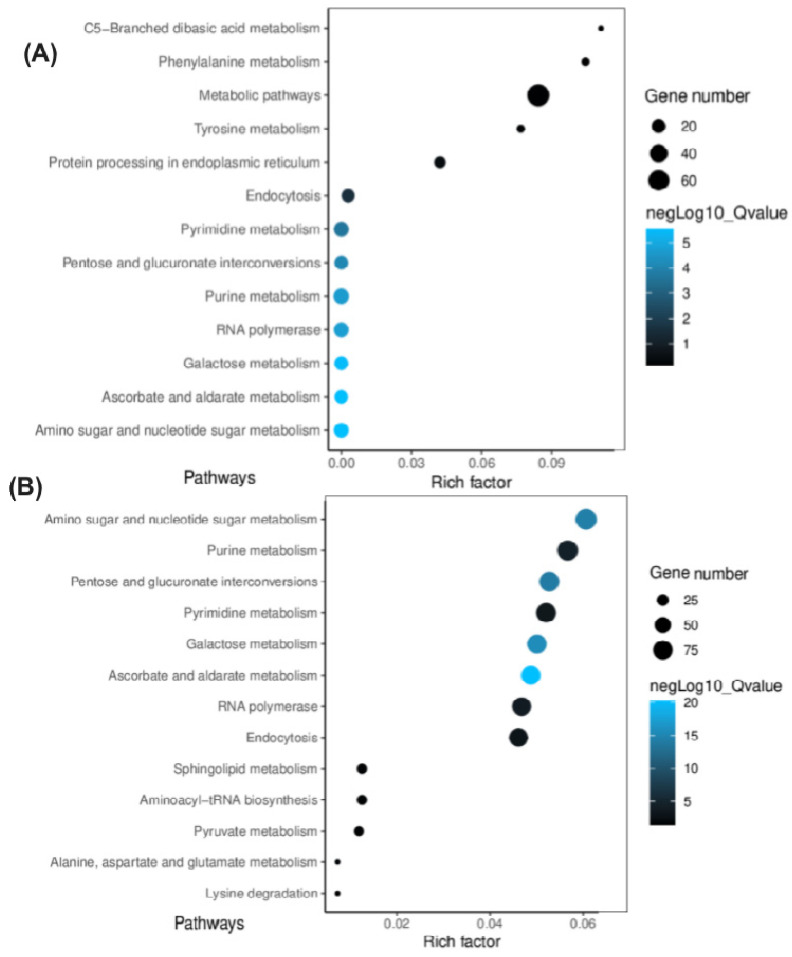
KEGG analysis for target genes of differently expressed miRNAs in response to salinity stress in the roots of Suntop (**A**) and Sunmate (**B**). The Y axis identifies the KEGG pathway, the X axis shows the enrichment ratio between the number of DEGs and all genes enriched in a particular pathway. The color of the dot represents the q-value, and the size of the dot represents the number of DEGs mapped to the referent pathway.

Using their default parameters, the TAPIR and TargetFinder toolboxes were used to predict target genes of DE miRNAs and to determine their possible functions under salinity stress. In some cases, there was no predictable target with three or fewer mismatches. Therefore, the best match was considered as the putative target. In total, 913 putative target genes were regulated by 90 miRNAs, of which 861 putative target genes were associated with 75 known miRNAs, and the remaining 52 putative target genes were associated with 15 novel miRNAs. To help determine the function of the miRNA targets and their association with any biological pathway, the Gene Ontology (GO) and Kyoto Encyclopedia of Genes and Genomes (KEGG) databases were used. Target genes were broadly classified into three primary GO domains: biological processes (BP), cellular components (CC), and molecular function (MF). Respectively, 320 and 1043 genes were predicted for Suntop and Sunmate, of which 37, 44, and 19% of these genes in Suntop and 52, 33, and 15% in Sunmate were placed in the BP, CC, and MF domains (Figure S2A,B). The genes in the GO domains were further grouped into different sub-categories (terms), with the CC domains containing seven and eight terms for Suntop and Sunmate, respectively. The most abundant terms were organelle, cell, and cell part for both cultivars, but the numbers of transcripts were higher for Sunmate than for Suntop. In the BP, 11 and 16 sub-categories (terms) were identified for Suntop and Sunmate, respectively. Genes responsible for regulation of biological, metabolic, and cellular processes were prominently expressed in both cultivars. In the MF domain, for both cultivars, most genes were associated with in the term binding; the number of expressed transcripts was higher in Sunmate than in Suntop. KEGG enrichment results for both control and salinity treatment were filtered by *p*-value, and the number of putative genes enriched in the first 20 entries with the smallest *p*-value (<0.05) were further analyzed. There were nine pathways (RNA polymerase, pyrimidine metabolism, purine metabolism, pentose and glucuronate inter-conversions, non-homologous end-joining, galactose metabolism, endocytosis, ascorbate and aldarate metabolism, and amino sugar and nucleotide sugar metabolism) (Figure 7A,B). For Suntop, metabolic pathways and C5 branched dibasic acid metabolism contained the least and highest rich factor (number of target genes of the specific pathway/total number of target genes mapped to this pathway) under the salinity treatment.

## 3. Discussion

Plants deploy several strategies to combat salinity stress. In general, molecular aspects of salinity tolerance are involved in modifying the transcription of a large number of genes resulting in the re-programming of plant physiology [34]. As known from previous studies, plant miRNAs play important roles in regulating stress-related gene expression [12,13,24]. Many scientists have worked with plants such as *Arabidopsis*, rice, barley, and maize to identify the numerous miRNAs produced due to various environmental stresses [1,12,14,26,35]. Here, a comprehensive study was conducted using two wheat cultivars, Suntop and Sunmate, that differ in salinity tolerance to identify the miRNAs and their putative targets associated with responses to salinity stress.

### 3.1. Effect of Salinity on Total ATPase Activity, Total Amino Acids and Soluble Sugar Contents

The foremost consequence of salinity stress is the generation of osmotic stress due to the low osmotic potential of saline rich solutions that restrict plant water uptake and leads to a loss of turgidity and dehydration of cells [36]. A common way plants protect themselves from osmotic stress is the production of compatible solutes, such as amino acid, soluble sugars, or other osmoprotectants [37]. In this study, salinity treatment of the tolerant and sensitive wheat cultivars resulted in contrasting levels of soluble sugars and amino acids. Suntop (salt-tolerant) showed a greater enhanced accumulation of both these osmolytes, a commonly observed response of tolerant cultivars under salinity stress, in comparison with Sunmate (salt-sensitive) in both the roots and leaves, suggesting that the sensitive cultivar was unable to deploy as effective a protection mechanism as the tolerant one.

In the present study, 191 candidate miRNAs produced in response to salinity stress were identified and consisted of 110 known and 81 novel miRNAs; 181 of these miRNAs were shared by the two cultivars. We systematically annotated the known miRNAs and showed that they belong to 35 families (Table S1) and, in addition, identified 12 wheat-specific families. This significantly increased the number of known miRNAs produced by wheat. Well-conserved miRNAs have homologous target interactions and control similar molecular functions among species. The most conserved miRNAs (miR156, miR159, miR164, miR166, miR167, miR169, miR171, miR172, miR319, and miR396) affect the levels of various families of transcription factors such as homeodomain-leucine zipper; auxin response factors (ARFs); myeloblastosis; squamosa-promoter binding protein-like; teosinte branched 1-cycloidea-PCF; APETALA2-like factors; *Arabidopsis* transcription activation factor (ATAF) and scarecrow-like subunits [38]. Such regulatory mechanisms are essential, because most of the miRNAs function through a complex regulatory network to coordinate plant development and stress responses [39].

### 3.2. tae-miR156, tae-miR160 and tae-miR171a/b May Confer Salt Tolerance in Roots of Suntop

Under salinity stress, the tolerant wheat cultivar, Suntop, down-regulated the expression of miR156, which negatively regulate its target squamosa promoter-binding like protein 3 (SPL3). Previously, the roles of *SPL* family genes as plant-specific transcription factors have been identified in several plant species including *A. thaliana*, rice, maize, *Brassica napus*, and soybeans [35,40,41,42,43]. During plant growth and development, SPL family genes are involved in several biological processes and underlie numerous vital agronomic characters in crops [44,45]. MicroRNA160, which targets auxin-responsive factor 8-like protein (ARF8), is one of the most conserved and abundantly expressed miRNAs in crops, having crucial roles in plant growth and development but also in response abiotic stresses [46,47]. In our study, miRNA160 was down- and up-regulated in Suntop and Sunmate, respectively, under salt stress, which is co-related with negatively regulation of ARF8. Furthermore, Ludwig-Müller [48] noted that the auxin response factor 8-like protein has a role in the transcriptional activation of genes in the auxin-responsive *GH3* (*GRETCHEN HAGEN 3*) gene family that are involved in the formation of auxin-conjugating proteins (IAA amino acid conjugates) thereby controlling free cellular levels and maintaining auxin homeostasis. Two other miRNAs, tae-miR171a and tae-miR171b, targeting scarecrow-like protein 6 (SCL6) putative transcription factors were suppressed in Suntop but induced in Sunmate. SCL6 is involved in developmental processes, including radial patterning in roots and hormone signaling [49].

### 3.3. miR319/PCF5 Module May Regulate Salt Tolerance in Suntop

PROLIFERATING CELL FACTORS (PCF) is a group of small transcription factors that have a function in regulating diverse plant growth and development processes by controlling cell proliferation. It was found that overexpression of miR319 conferred salt tolerance in rice by regulating several physiological pathways [50]. However, the function of miR319 and its target PCF5 gene and the mechanism of miR319-PCF5 module-enhanced plant resistance to salt stress in wheat have been little studied. In this study, we demonstrated that the miR319 target PCF5 gene might enhance salt tolerance in Suntop. Overexpressing of miR319 in transgenic lines enhanced the regulation of potassium (K^+^) transporter genes such as HKT, AKT, and HAK, ultimately enhancing K^+^ accumulation and a higher K^+^/Na^+^ ratio in OE-miR319 lines in switchgrass [51]. It is well known that under salinity stress, K^+^ helps in osmotic adjustment and so help salt tolerance [52,53]. In our previous studies, compared with Sunmate, we found a higher K^+^ concentration, a lower Na^+^/K^+^ ratio in Suntop [28] and induced levels of *Ta*HKT and *Ta*AKT genes [54]. Zhou et al. [50] reported that in creeping bent grass miR319 enhances salt tolerance by altering leaf cuticular waxes and root length. Furthermore, besides higher salt tolerance, the miR319/PCF5 module also enhances biomass in switchgrass [51].

### 3.4. miR159a-b, and miR9657 May Regulate Salt Tolerance in Suntop

The transcription factor, R2R3 MYB, is a well-known role in the regulation of stress responses and plant growth in many plant species, for instance, MYB4 is involved in defense against salinity in alfalfa [55,56]. *Ms*MYB2L is also rapidly induced by NaCl, ABA, and mannitol in tolerant cultivars, suggesting that MYBs are involved in stress tolerance [57]. Similarly, *Bna*MYB21, *Bna*MYB141, and *Bna*MYB148 are involved in the regulation of salt tolerance [58]. Our results show that the regulation of miR159a-b and miR9657 was suppressed in Suntop, thereby enhancing the expression of their respective target genes that encode the MYB-related transcription factors. Previously, in *Arabidopsis,* it was found that *Ms*MYB2L increase the expression of several genes belonging to the ABA-dependent pathway under salinity stress; it is likely that the same applies for the MYB in wheat [57]. Furthermore, OE-*Gm*MYB68 promoted the accumulation of soluble sugars and proline in soybean under salinity, indicating that MYBs might help in osmotic pressure adjustment at cellular level [59]. Consistent with this, we found greater soluble sugars in Suntop (Figure 1). Taken together, this suggests that MYB-related transcription factors have a role in salt stress tolerance.

### 3.5. Novel miRNAs Exclusively Expressed in Suntop

In the present study, we also identified novel miRNAs that may have roles in salt tolerance in Suntop. The expression of novel-mir59 that targets two genes, *Traes*CS6B01G465600.1, and *Traes*CS6B01G465600.2, encoding CBL-interacting serine/threonine-protein kinase 7-like was unchanged in Suntop but up-regulated in Sunmate. CBL proteins [60,61] interact with a group of serine/threonine protein kinases, CIPKs, that are associated with plant responses to environmental stresses [62] CIPK7, a member of this group, maybe a candidate, downstream target of one or more CBLs, and Huang et al. [62] found that in vitro CIPK7 interacted with CBL1. Similarly, Kolukisaoglu et al. [61] reported that *At*CBL1 exhibited a significant interaction with CIPK7 in a yeast two-hybrid analysis. Previous studies have deduced that CIPKs are involved in reactions to abiotic stresses. For example, CBL10–CIPK24 and CBL4–CIPK24 modules mediated salt tolerance in the shoots and roots of *Arabidopsis* [63] by interacting with a vacuolar membrane-localized CBL protein. Therefore, CIPK24 could play a role in stimulating vacuolar Na^+^/H^+^ exchange [64]. Similarly, the *Ta*CBL3–*Ta*CIPK29 complex regulates the antioxidant system, and transporter genes protect wheat from salt stress [65]. So, taken together, we can speculate that novel-mir59 and its potential targets (CBL-interacting serine/threonine-protein kinase 7-like) also have a role in salt tolerance in Suntop.

## 4. Materials and Methods

### 4.1. Plant Materials and Experimental Design

A growth room hydroponic experiment was carried out at the Zijingang Campus, Zhejiang University, Hangzhou, China, in 2019. Two wheat cultivars, Suntop (salt-tolerant) and Sunmate (salt-sensitive) [28,55] were used in this study. Seeds were treated with 3% H_2_O_2_ for 10 min, rinsed seven times with distilled water, before sowing in wet sand. The seeds were sown in a controlled environment with a day-night temperature of 22 ± 3 °C, a relative humidity of 75%, and 8 h of light with an intensity of 250 μmol m^−1^ × s^−1^ of PAR. Ten days after germination, uniform seedlings were transferred to 2 L pots containing 1.5 L basic nutrient solution (BNS). The container was covered with a polystyrol-plate with five evenly spaced holes with two plants per hole. On the seventh day after transplanting, Na (as NaCl) was added to the containers to form two treatments: BNS (control) and BNS plus 100 mM NaCl. The experiment had a split-plot design with treatment as the main plot and cultivar as the sub-plot, and there were four replicates for each treatment. The composition of BNS was (mg L^−1^): (NH_4_)_2_SO_4_, 48.2; MgSO_4_, 65.9; K_2_SO_4_, 15.9; KNO_3_, 18.5; Ca(NO_3_)_2_, 59.9; KH_2_PO_4_, 24.8; Fe-citrate, 5; MnCl_2_ 4H_2_O, 0.9; ZnSO_4_ 7H_2_O, 0.11; CuSO_4_ 5H_2_O, 0.04; HBO_3_, 2.9; H_2_MoO_4_, 0.01. The solution pH was adjusted to 5.8 ± 0.1 with NaOH or HCl, as required.

Plant roots samples for RNA isolation were collected 1 d after treatment, while plant roots and leaves samples for other biochemical traits were collected 5 d after treatment. All samples were stored at −80 °C for downstream analysis.

### 4.2. Determination of Total Soluble Sugars

Soluble sugar content was measured using the spectrophotometric method described by Zhang et al. [66]. Dry samples of wheat leaves were boiled in distilled water for 30 min. The extracts were filtered through two layers of cheesecloth. The filtrate (0.5 mL) was mixed with 1.5 mL distilled water and 1 mL of 9% phenol, and then 5 mL H_2_SO_4_. Tubes containing this mixture were incubated at room temperature for 30 min. The color change was estimated using a spectrophotometer at 485 nm. The soluble sugar contents were determined against a standard curve prepared using glucose.

### 4.3. Determination of Total Amino Acids Contents and ATPase Activity

Total amino acids OD values for all samples were measured at 650 nm using the total amino acid (T-AA) assay kit (Jiancheng Bio Co., Nanjing, China). Total soluble protein was calculated spectrophotometrically using bovine serum albumin as a standard [67]. ATPase activity was determined using an activity assay kit (Jiancheng Bio Co., Nanjing, China).

### 4.4. Small RNA Library Construction and High Throughput Sequencing

Total RNA was extracted from wheat roots using TRIzol reagent (Takara Co., Tokyo, Japan) following the manufacturer’s instructions. The extracted total RNA was treated with DNase I to remove any contaminating genomic DNA. RNA purity was checked using an Agilent 2100 bioanalyzer (Agilent Technologies, CA, USA), while integrity of the RNA was visualized using polyacrylamide gels. Small RNA segments ranging from 18–30 nt were separated by PAGE gel for sRNA libraries and then ligated to 5′- and 3′- adapters. Using the Superscript II reverse transcriptase (Invitrogen, CA, USA), each RNA sample was reverse transcribed and then amplified by PCR. High throughput sequencing of small RNAs was established using BGISeq 500 technology at BGI-Hangzhou, China.

### 4.5. Data Pre-Processing and De Novo Assembly

The impurities in the raw read such as 5′ contamination, no inset tags, low-quality tags, poly-A tag, small tags and tags without 3′ primer were removed using ACGT101- miR (LC Science, Houstan, TX, USA) according to the software handbook. Only sRNA sequences of 18 to 30 nts were kept for downstream analysis. The clean tags were aligned to mRNA wheat genome shotgun sequence assemblies (http://mips.helmholtz-muenchen.de/plant/wheat/uk454survey/index.jsp, accessed on 18 October 2021) [68] while the cmsearch tool was used to search for Rfam alignment (including rRNA, tRNA, snRNA, and snoRNA, etc. version 11; http://rfam.janelia.org, accessed on 18 October 2021). Also, the complete set of aligned reads were compared against siRNA, rRNA, tRNA, phasiRNA5S, and NOR RNA loci in the genome, and all reads that aligned by at least 90% with any of these was categorized as such using BEDTools intersect. All remaining reads were categorized as miRNAs reads and used for subsequent miRNAs analyses. Subsequently, the clean sequences were subjected to BLASTN searches against miRBase (http://www.mirbase.org, accessed on 18 October 2021) using Bowtie2 (parameters; ‘-v 2 -best -strata -m 1’) to obtain mature miRNAs alignments with up to two mismatches [69]. The conserved microRNAs in wheat were compared with other plant species to explore their evolutionary conservation The software package miRA [70] was used to predict novel miRNAs by exploring the characteristic hairpin structure of the miRNA precursor.

### 4.6. Differential Expression of Salinity-Responsive miRNAs

Potentially expressed wheat miRNAs were analyzed to determine the following: (1) there was at least 100 transcripts per million (TPM) putative miRNAs in one of the four libraries; (2) the length of miRNAs was 20 to 24 nt; (3) the counts for 5p and 3p could be evaluated and the ratio of 5p/3p or 3p/5p was higher than 0.9; (4) the precursor miRNA minimum folding free energy was lower than −37 kJ/mol; (5) the minimal free energy index (MFEIs) was higher than 0.85; and (6) hairpin secondary structure for pre-miRNA was predicted by RNA fold. The following formula was used to calculate the TPM:

(1) $$\mathrm{TPM}=\frac{C*10^{6}}{N}$$

where *C* is the miRNA count in a sample and *N* is the total reads number that mapped to the genome.

Later an analysis software, DEGseq [71], based on a negative binomial distribution was used to analyze differentially expressed miRNAs between control vs. salinity treatment for each group. The fold change between salinity treatment and control normalized reads in Suntop and Sunmate was calculated using the formula: fold change = log_2_ (salinity reads/control reads). miRNAs with fold change log_2_ N ≥ 1 were considered as up-regulated, between 0 < |log_2_ N| < 1 were considered as unchanged and log_2_ N ≤ −1 were considered as down-regulated.

### 4.7. Target Gene Prediction and GO and KEGG Enrichment Analysis

In order to predict target transcripts, we used multiple types of software such as TAPIR and TargetFinder with default parameters to predict wheat target genes. GO enrichment analysis was carried out to determine which functional modules, such as biological, cellular, and/or molecular, contained the highest numbers of target genes. The Gene Ontology (GO) database (http://www.geneontology.org/, accessed on 18 October 2021) R package was used to perform GO enrichment analysis for the target genes. Firstly, *p* values were corrected using the Bonferroni method, the corrected *p* value *<* 0.05 was considered as a threshold, and the GO terms with these criteria were defined as significantly enriched GO terms. To get into a more depth assessment of the metabolic pathways, we carried out KEGG pathway enrichment analysis of the target genes (http://www.genome.jp/eg/, accessed on 18 October 2021). For hierarchical cluster analysis of gene expression patterns, the software used was Cluster and Java Tree View, respectively.

### 4.8. mRNAs and miRNAs Validation by qRT-PCR

Randomly selected potential mRNAs and miRNAs were used to validate the sequencing data. The RNA samples used for cDNA synthesis were the same as those used for small RNA library construction. First-strand cDNA for targeted mRNA genes were synthesized using a PrimeScript RT reagent kit with a genomic DNA eraser (Takara, Tokyo, Japan), while Mix-XTM miRNA first-strand synthesis kit was used to generate miRNA cDNA (Takara, Tokyo, Japan). Later, using SYBR Advantage^®^ qPCR Premix (Takara, Tokyo, Japan), the assay was performed with quantitative real-time PCR (qRT-PCR) in a Lightcycler480 (Roche International Diagnostics Systems, Rotkreuz, Switzerland). MicroRNA specific primers were designed with primer premier5 software. The entire mature sequence (21–23 nts) of the miRNA was used as miRNA specific, 5′ primer while the 3′ primer for qPCR was the mRQ 3′ Primer supplied with the kit. Three biological replicates and two technical replicates were carried out for each mRNA. The beta-actin gene (F-CACTGGAATGGTCAAGGCTG; R-CTCCATGTCATCCCAGTTG) for mRNAs and U6 for miRNAs were used as internal control/reference using comparative Ct methods. The relative expression changes were calculated using the 2−∆∆Ct method. All primers are listed in the Tables S7 and S8.

### 4.9. Statistical Analysis

Pooled data were subjected to ANOVA analysis using SPSS software (Statistical Program for Social Science), following Duncan’s Multiple Range Tests (DMRT) to distinguish between the means at *p* ≤ 0.05.

## 5. Conclusions

**Figure 8:**
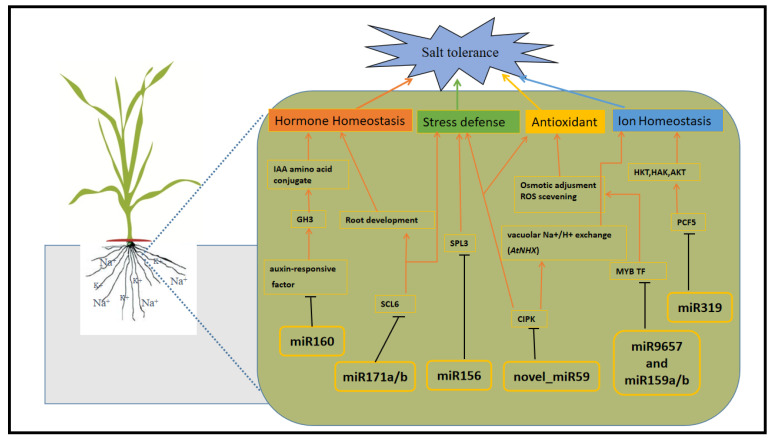
A model representing NaCl tolerance in Suntop. All miRNAs are either unchanged or down-regulated in Suntop. ARF3 (Auxin responsive factor 3), CIPK (CBL-interacting protein kinases), IAA (Indole-acetic acid), GH3 (GRETCHEN HAGEN 3), PCF5 (Proliferating cell factors), SCL6 (Scarecrow-like protein 6), SPL3 (Squamosa promoter binding like protein 6).

In the present study, microRNAs and putative target genes expression patterns of salt-tolerant Suntop and salt-sensitive Sunmate in responses to salinity were analyzed by high throughput sequencing. In total, 110 known and 81 novels miRNAs were identified belonging to 35 families, among them, 181 miRNAs were shared by both cultivars. Upon salinity stress, the numbers of differentially expressed miRNAs found were 43 and 75 in Suntop and Sunmate, respectively. We propose a model based on the known and novel miRNAs involved in molecular mechanisms of salt tolerance in the roots of Suntop (Figure 8). Suntop showed high salt tolerance by modifying the regulation of tae-miR156, tae-miR160, tae-miR171a-b, tae-miR319, tae-miR159a-b, tae-miR9657 and novel_mir59 which mainly regulate auxin responsive factor (ARF), SPL, SCL6, PCF5, R2R3 MYB, and CBL-CIPK which may be involved in cell growth, ion homeostasis, hormone signaling, redox maintenance, and stress defense. This work enhances our understanding of the underlying regulatory processes and the role of miRNAs and their target genes in the salt tolerance of Suntop. Further in vivo and in vitro studies are needed to gain further insights into the associations and the regulatory responses between miRNAs and their predicted target genes, biological pathways, and molecular network in wheat.

## Figures and Tables

**Table 1:** Summary of high-throughput sequencing of small RNAs from the roots of the two wheat cultivers Suntop and Sunmate.

Library Type	Suntop	Suntop	Sunmate	Sunmate
	Control	NaCl	Control	NaCl
Total raw reads	29,114,838.5	28,580,752.5	28,973,938.5	29,753,197
Total clean reads	27,175,593.5	26,292,555	25,900,067	25,819,464
Clean tag (%)	95.08	90.31	89.39	86.77
Q20 of clean tag (%)	99.20	99	99.30	99.30
Mapped reads	24,908,414 (91.66%)	24,352,075.5 (92.62%)	24,230,503.5 (93.55%)	22,175,264 (85.89%)
Total miRNAs reads	4,180,023	5,014,878.5	5,127,462.5	5,644,374.5
Known miRNAs reads	529,287	452,384.5	627,551	242,607
Novel miRNAs reads	3,650,736	4,562,494	4,499,911.5	5,401,767.5

## Data Availability

All data supporting the reported results are included within the article or its Supplementary Materials Files.

## Supplementary Materials

The following are available online at https://www.mdpi.com/article/10.3390/ijms222212556/s1. Figure S1. Numbers of miRNAs in two wheat cultivars under NaCl stress (T) and control conditions (CK) in roots (A). Length base distribution of total identified miRNAs in roots of two wheat cultivars (B) Suntop (ST), (C) Sunmate (SM); Figure S2. GO analysis of identified target genes in Suntop (A) and Sunmate (B); Table S1. Description, expression and putative target genes of known and novel miRNAs in roots whose expression were significantly down-regulated or up- regulated in of both Suntop (ST), and Sunmate (SM) treated with 100 mM NaCl for 1 day; Table S2. Evolutionary conservation of known miRNA families in other plant species; Table S3. Description, expression and putative target genes of known miRNAs in roots whose expression were unchanged in Suntop (ST) but up-regulated in Sunmate (SM), or down-regulated in ST but up-regulated or unchanged in SM treated with 100 mM NaCl for one day; Table S4. Description, expression and putative target genes of known miRNAs in roots whose expression were unchanged in Suntop (ST) but down-regulated in Sunmate (SM), or up-regulated in ST but down-regulated or unchanged in SM treated with 100 mM NaCl for one day; Table S5. Description, expression and putative target genes of novel miRNAs in roots whose expression were unchanged in Suntop (ST) but up-regulated in Sunmate (SM), or down-regulated in ST but up-regulated (-) or unchanged in SM treated with 100 mM NaCl for one day; Table S6. Description, expression and putative target genes of novel miRNAs in roots whose expression were unchanged in Suntop (ST) but down-regulated (-) in Sunmate (SM), or up-regulated in ST but down-regulated or unchanged in SM treated with 100 mM NaCl for one day; Table S7. List of gene specific primers used in qRT-PCR analyses; Table S8. List of miRNA primers used in qRT-PCR analyses.
