# Supplementary material for: Genome-Wide Discovery of miRNAs with Differential Expression Patterns in Responses to Salinity in the Two Contrasting Wheat Cultivars
Source: Int J Mol Sci. 2021 Nov 21;22(22):12556. doi: 10.3390/ijms222212556 (PMC8621374; doi:10.3390/ijms222212556)
Supplement: Supplementary file 1 [file ijms-22-12556-s001.zip › ijms-1447656-supplementary.pdf]

Supplemental Material

Genome-wide discovery of miRNAs with differential expression patterns in responses to salinity in the two contrasting wheat cultivars

Muhammad Zeeshan[1], Cheng-Wei Qiu[1], Shama Naz[1], Cao Fangbin[1*], Wu Feibo[1,2*]

[1]Department of Agronomy, College of Agriculture and Biotechnology, Zijingang Campus, Zhejiang University, Hangzhou 310058, P.R. China 11616102@zju.edu.cn (M.Z.); 3130100260@zju.edu.cn (C.Q.); shamaktk@yahoo.com (S.N.); wufeibo@zju.edu.cn (W.F.)

[2]Jiangsu Co-Innovation Center for Modern Production Technology of Grain Crops, Yangzhou University, Yangzhou 225009, China

*Corresponding author: wufeibo@zju.edu.cn; Tel./Fax: +86 571 88982827

Table S1. Description, expression and putative target genes of known and novel miRNAs in roots whose expression were significantly down-regulated or up- regulated in of both Suntop (ST), and Sunmate (SM) treated with 100 mM NaCl for 1 day.

Family name	miRNA name	Sequence	Length (nt)	Fold change [1]		Target gene	Annotation
				ST	SM		
MIR156	tae-miR156	UGACAGAAGAGAGUGAGCACA	21	-1.28	-0.73	TraesCS2A01G350100.1	Nitrate transporter 1.2
						TraesCS2B01G368600.1	
						TraesCS2B01G368600.2	
						TraesCS2D01G348500.1	protein NRT1/ PTR FAMILY 4.6-like isoform X1
						TraesCS2D01G348500.2	
						TraesCS2D01G348500.3	
						TraesCS6A01G110100.2	squamosa promoter-binding-like protein 3
						TraesCS6B01G138400.1	
						TraesCS6D01G098500.1	
						TraesCS6D01G145200.1	squamosa promoter-binding-like protein 4
MIR159	tae-miR159a, tae-miR159b	UUUGGAUUGAAGGGAGCUCUG	21	-0.39	1.93	TraesCS3A01G131700.1	protein SPEAR2
						TraesCS3A01G131700.3	
						TraesCS3D01G147900.1	
						TraesCS3D01G147900.2	
						TraesCS3A01G336500.1	transcription factor GAMYB
						TraesCS3D01G329400.1	
						TraesCS3D01G329400.2	
						TraesCS3D01G329400.3	
						TraesCS3B01G367500.1	R2R3-MYB protein
						TraesCS3B01G367500.2	
MIR160	tae-miR160	UGCCUGGCUCCCUGUAUGCCA	21	-1.58	1.42	TraesCS2A01G380300.1	auxin response factor 8-like

Family name	miRNA name	Sequence	Length (nt)	Fold change [1]		Target gene	Annotation
				ST	SM		
						TraesCS2B01G397300.1	
						TraesCS4B01G346200.1	protein FIZZY-RELATED 2-like
						TraesCS4B01G346200.2	
						TraesCS4D01G341200.1	
MIR167	tae-miR167a	UGAAGCUGCCAGCAUGAUCUA	21	-0.82	1.62	TraesCS3B01G253400.1	hypothetical protein TRIUR3_14845
	tae-miR167c-5p	UGAAGCUGCCAGCAUGAUCUGC	22	-1.02	-0.66	TraesCS3D01G275100.2	hypothetical protein F775_08516
MIR171	tae-miR171a	UGAUUGAGCCGUGCCAAUAUC	21	-2.46	1.46	TraesCS1A01G209400.1	scarecrow-like protein 6
						TraesCS1B01G223400.1	
						TraesCS1D01G212800.1	
	tae-miR171b	UUGAGCCGUGCCAAUAUCACG	21	-1.03	-0.89	TraesCS6A01G247200.1	scarecrow-like protein 6
						TraesCS6B01G277400.1	
						TraesCS6D01G229400.1	
						TraesCS2A01G352600.1	6,7-dimethyl-8-ribityllumazine synthase, chloroplastic
						TraesCS2B01G371700.1	
						TraesCS2D01G351600.1	
	tae-miR319	UUGGACUGAAGGGAGCUCCCU	21	-0.21	-2.97	TraesCS3D01G146900.1	transcription factor PCF5-like
						TraesCS3A01G140100.1	
						TraesCS7A01G176600.1	chaperone protein dnaJ 1, mitochondrial isoform X1
						TraesCS7B01G081600.2	
						TraesCS7D01G177900.1	
MIR395	tae-miR395a	GUGAAGUGUUUGGGGGAACUC	21	-0.45	3.49	TraesCS5B01G387300.1	ATP sulfurylase 4, chloroplastic-like
						TraesCS5D01G392300.1	
	tae-miR395b	UGAAGUGUUUGGGGGAACUC	20	-1.29	-0.16	TraesCS5A01G382900.1	ATP sulfurylase

Family name	miRNA name	Sequence	Length (nt)	Fold change [1]		Target gene	Annotation
				ST	SM		
MIR397	tae-miR397-5p	UCACCGGCGCUGCACACAAUG	21	1.76	-0.69	TraesCS7A01G047900.1	probable protein phosphatase 2C 37
MIR398	tae-miR398	UGUGUUCUCAGGUCGCCCCCG	21	-1.8	-1.2		
undef	tae-miR1117	UAGUACCGGUUCGUGGCACGAACC	24	-0.32	-1.47		
undef	tae-miR1125	AACCAACGAGACCAACUGCGGCGG	24	0.28	-2.41		
MIR818	tae-miR1130b-3p	UCUUAUAUUAUGGGACGGAGG	21	-0.22	1.09	TraesCS2D01G248200.6	probable inactive leucine rich repeat receptor-like protein kinase At3g03770 isoform X2
	tae-miR1137a	UAGUACAAAGUUGAGUCAUC	20	0.25	-1.53	TraesCS6A01G107500.1	unnamed protein product
						TraesCS6B01G136100.1	
						TraesCS6B01G136200.1	
						TraesCS2A01G366400.1	hypothetical protein OsI_25001
						TraesCS2A01G366400.2	hypothetical protein F775_04208
Undef	tae-miR5048-5p	UUUGCAGGUUUUAGGUCUAAGU	22	-0.72	1.24	TraesCS3A01G521100.1	uncharacterized protein
						TraesCS3A01G521100.2	LOC109783670
						TraesCS4B01G313900.2	
						TraesCS4B01G313900.3	
						TraesCS1B01G119300.3	starch synthase III
						TraesCS7D01G487800.1	GDSL esterase/lipase
MIR5062	tae-miR5062-5p	UGAACCUUAGGGAACAGCCGCAU	23	-0.77	1.34		
MIR5067	tae-miR5175-5p	UUCCAAAUUACUCGUCGUGGU	21	-0.32	1.4	TraesCS2A01G485700.2	receptor-like serine/ threonine -protein kinase SD1-8
MIR5200	tae-miR5200	UGUAGAUACUCCCUAAGGCUU	21	-0.09	1.2	TraesCS5D01G072900.1	hypothetical protein F775_12042
MIR7757	tae-miR7757-5p	AUAAAACCUUCAGCUAUCCAUC	22	-1.1	1.78	TraesCS2A01G023400.1	putative disease resistance protein
						TraesCS2B01G033500.1	At1g50180

Family name	miRNA name	Sequence	Length (nt)	Fold change [1]		Target gene	Annotation
				ST	SM		
						TraesCS2D01G024500.1	
						TraesCS2D01G024500.2	
MIR9652	tae-miR9652-3p	AAGCUUAAUGAGAACAUGUG	20	1.24	0		
	tae-miR9652-5p	CCUGUUUGUCAUUAAGUUUCUU	22	-0.54	1.41	TraesCS3B01G287600.2	protein argonaute MEL1
						TraesCS5B01G451400.1	
						TraesCS5B01G452500.1	
Undef	tae-miR9653b	UGGCCAAGGUCUCUUGAGGCU	21	-1.07	1.8	TraesCS1A01G228200.1	probable protein phosphatase 2C 48
						TraesCS1A01G228200.2	
MIR9654	tae-miR9654b-3p	UUCCGAAAGGCUUGAAGCGAAU	22	-0.9	2.18		
Undef	tae-miR9656-3p	CUUCGAGACUCUGAACAGCGG	21	-0.17	1.72		
MIR9657	tae-miR9657a-3p	UGUGCUUCCUCGUCGAACGGU	21	-0.31	1.27	TraesCS3B01G280400.1	WD-40 repeat-containing protein
						TraesCS3D01G251300.1	MSI4 isoforms
						TraesCS3A01G250900.2	
						TraesCS7B01G144300.1	MYB-related protein
						TraesCS7B01G006500.1	putative serpin-Z5
						TraesCS7D01G103400.1	
						TraesCS3A01G250900.3	FVE [Triticum aestivum]
						TraesCS3A01G250900.1	
	tae-miR9657c-3p,	CGUGCUUCCUCGUCGAACGGU	21	-0.38	1.16	TraesCS3B01G280400.1	WD-40 repeat-containing protein
	tae-miR9657b-3p					TraesCS3D01G251300.1	MSI4 isoforms
						TraesCS3A01G250900.2	
						TraesCS7B01G006500.1	putative serpin-Z5
						TraesCS7D01G103400.1	
						TraesCS7B01G144300.1	MYB-related protein

Family name	miRNA name	Sequence	Length (nt)	Fold change [1]		Target gene	Annotation
				ST	SM		
						TraesCS3A01G250900.1	FVE [Triticum aestivum]
						TraesCS3A01G250900.3	
undef	tae-miR9658-3p	AUCGUUCUGGGUGAAUAGGCC	21	-0.25	-2.03		
MIR9662	tae-miR9662a-3p	UUGAACAUCCCAGAGCCACCG	21	1.12	1.19	TraesCS4B01G290700.1	transcription termination factor MTERF9, chloroplastic-like
						TraesCS5D01G030400.1	transcription termination factor MTEF18, mitochondrial-like
						TraesCS6A01G015200.1	transcription termination factor
						TraesCS6D01G036200.1	MTERF15, mitochondrial-like
						TraesCS6B01G025600.1	transcription termination factor MTERF4, chloroplastic-like
						TraesCS6B01G059500.1	transcription termination factor
						TraesCS6B01G059600.1	MTERF8, chloroplastic-like
						TraesCS7B01G292800.1	GDSL esterase/lipase At3g09930-like
	tae-miR9662b-3p	UGAACAUCCCAGAGCCACCGG	21	-0.09	1.45	TraesCS6B01G021800.1	Uncharacterized protein
						TraesCS6B01G043100.1	LOC109765037
						TraesCS6D01G035100.1	cytosolic sulfotransferase 5-like
undef	tae-miR9664-3p	UUGCAGUCCUCGAUGUCGUAG	21	-0.64	1.04	TraesCS3A01G247700.1	cyclin dependent protein kinase
						TraesCS7D01G096000.1	disease resistance protein
						TraesCS5D01G405600.1	
						TraesCS2D01G541000.1	
						TraesCS2D01G025700.1	
						TraesCS2D01G025700.2	

Family name	miRNA name	Sequence	Length (nt)	Fold change [1]		Target gene	Annotation
				ST	SM		
						TraesCS2D01G025700.3	
MIR9666	tae-miR9666a-3p	CGGUAGGGCUGUAUGAUGGCGA	22	-1.71	-0.69	TraesCS5B01G438300.1	BTB/POZ and MATH domain-containing protein 3
	tae-miR9666b-3p	CGGUUGGGCUGUAUGAUGGCGA		-2.18	-1.13		
	tae-miR9666c-5p, tae-miR9666b-5p	GCCAUCAUACGUCCAACCGUG	21	-2.43	-0.69	TraesCS2B01G562600.1	exocyst complex component EXO70A1-like
undef	tae-miR9668-5p	CCAAUGACAAGUAUUUUCGGA	21	-0.28	1.75	TraesCS6B01G070200.1	disease resistance protein RGA2-like
undef	tae-miR9669-5p	UACUGUGGGCACUUAUUUGAC	21	-0.72	2.13		
MIR9672	tae-miR9672a-3p	CCACGACUGUCAUUAAGCAUC	21	0.65	-1.02		
	tae-miR9672b	UACCACGACUGUCAUUAAGCA	21	-1.12	-0.57	TraesCS7D01G383200.1	replication factor C subunit 1
MIR9674	tae-miR9674b-5p	AUAGCAUCAUCCAUCCUACCC	21	-0.08	1.48	TraesCS2A01G531000.1	protein Rf1, mitochondrial-like
						TraesCS6A01G014800.1	
						TraesCS6B01G021600.1	
						TraesCS6D01G017700.1	
undef	tae-miR9675-3p	UUUAUGAUCACUCUCGUUUUG	21	-0.21	2.48	TraesCS3A01G074300.1	premnaspirodiene oxygenase like
MIR9772	tae-miR9772	UGAGAUGAGAUUACCCCAUAC	21	-0.58	1.7	TraesCS6A01G063700.1	F-box protein At5g03970 like
						TraesCS6B01G085300.1	
						TraesCS6B01G085300.2	
						TraesCS6D01G061100.1	
						TraesCS7D01G005400.1	F-box/kelch-repeat protein At3g61590-like
						TraesCS6A01G058900.1	
undef	tae-miR9773	UUUGUUUUUAUGUUAUUUUGUGA	23	-0.39	2.63		

Family name	miRNA name	Sequence	Length (nt)	Fold change [1]		Target gene	Annotation
				ST	SM		
Undef	tae-miR9774	CAAGAUAUUGGGUAUUUCUGUC	22	-1.09	-2.13		
Undef	tae-miR9775	UGUGCGCAAUAAGAUUUUGCUA	22	0.46	1.97	TraesCS7D01G005400.1	F-box/kelch-repeat protein SKIP11
Undef	tae-miR9778	UGCAUCAUCUCGAACUCGUCG	21	-0.62	-2	TraesCS1A01G021700.1<br>TraesCS1A01G021800.1<br>TraesCS1A01G021800.2<br>TraesCS1B01G025500.1<br>TraesCS1B01G027000.1<br>TraesCS1B01G027500.1<br>TraesCS1B01G027500.2	Disease resistance protein RPM1
	novel_mir3	AUUGUAGUCUGGAGAGGCGUCCUCA GCGAC	30	1.26	1.54		
	novel_mir8	UCCCGGCCCCGAACCUGUCGGCU	23	-0.37	1.17	TraesCS6A01G114800.1<br>TraesCS6B01G142400.1	premnaspirodiene oxygenase-like
	novel_mir9	UUUGAAGACUAGUUUAUUAUAU	22	-0.36	-2		
	novel_mir11	ACGCAUCAUUCAAAUUUCUGCCCUA UCA	28	-0.23	-1.42		
	novel_mir13	CCAUGGCCAAGGUCUCUUGAGGC	23	-2	-1.34		
	novel_mir14	AGAGAUCCCGCGCGCUACUCCGUCGA	26	-1.53	-0.49		
	novel_mir16	CUUGAAGACUUUGGCCACGUCCAU	24	-1.69	-0.68		
	novel_mir17	ACCUUUGAGACUUUGGCCAUGGCCA	25	-1.83	-0.94		
	novel_mir18	UUGAAGACUUUGGCCAUGUCCAUGG UG	27	-1.34	-0.99		
	novel_mir19	UCCCACAGUGCAAAUUUAAAUUUUA UUAGA	30	-0.45	-1.01		

Family name	miRNA name	Sequence	Length (nt)	Fold change [1]		Target gene	Annotation
				ST	SM		
	novel_mir23	AGCACCGUUGGCAUCAUCGUCGGCGUUGUC	30	-0.57	-1.16		
	novel_mir24	AUGGGCUACGUGUGUUAAAC	20	-2.34	-0.69		
	novel_mir25	UUGGGUUCGAGCCCCAAGGUGGG	23	1.69	0.98		
	novel_mir26	AGCACCGUUGGCAUCAUUGUCGGCGUUGUU		-0.47	-1.25		
	novel_mir27	CGCCUUGGGGCCCGAACCCAAGACCA	26	-1.91	-2.22		
	novel_mir28	ACUGGUUGGAUCAUGCUUCUGUUUAUGA	28	-0.87	1.38	TraesCS1D01G090600.1	putative disease resistance RPP13-like protein 3
						TraesCS6B01G387200.1	Disease resistance protein RPM1
	novel_mir29	AGUUCGAGUCGGAGGCCACGGUGCUGGG	28	-0.33	-1.47		
	novel_mir30	UUGUUUGGCCAUCAAGAACAAUAGUAG	27	-0.36	-1.32		
	novel_mir31	AUCGAGAAAUUGGAGCUCGGUGCAGGCAUA	30	-0.62	-1.39		
	novel_mir32	UGUUUGGUGCGGACUCUGGACCU	23	-1.23	-0.96		
	novel_mir33	AAGAACAUCUAAGGGGCUGAGUUG	24	-1.56	-0.23		
	novel_mir34	AAAAUGAUGUGUUGUGGAAAGCA	23	-1.91	1.84		
	novel_mir36	UUGGCGAUAGCGAAUGCAGUUCUC	24	-2.31	-0.16		
	novel_mir37	AAGUUGCGUAGUGGAUCGCUUGGGGCCUA	29	-0.78	-1.18		
	novel_mir39	GACGGAAGGAUUUGGCGGGACCG	23	-4.09	-3.07		

Family name	miRNA name	Sequence	Length (nt)	Fold change [1]		Target gene	Annotation
				ST	SM		
	novel_mir40	GUGCACCCGUGUUCACCUUUGUAUUU	26	-0.28	1.93		
	novel_mir42	UUGAACCUUGGGGAAAAGCCGCAUA	25	-0.89	1.42		
	novel_mir45	UGAUUAACGGCCAGGAUUUCCCUG	24	-0.13	1.12		
	novel_mir46	GCGGCGACGGGGGCGGCUU	19	3.21	-1.39		
	novel_mir47	GCGAGAGCGGGUCGCCGCGUGCCG	24	-1.35	-1.75	TraesCS4B01G303700.1	hypoth protein TRIUR306439
	novel_mir48	AUGGAUCUGACGGCUGUAGAGGA	23	-0.43	1.16		
	novel_mir49	GUGAUUAACGGCCAGGAUUUCCCUG	26	-2.18	1.69		
	novel_mir51	GAUGAUAUCUAUAUUAUCACUCUGAGGGA	29	-1.21	-1.58		
	novel_mir52	CCCGUGGAACUUUCUUUUUGAAAA	24	-9.75	-8.5		
	novel_mir53	CCGGAUGUGUUGUCUUCCUCGACCAUGGUG	30	-0.01	-1.3		
	novel_mir54	UUUGGGGGAUCGAAGAUGAUUAAA	24	-2.91	-2.77		
	novel_mir55	GGUGGUUGCUGGCGCGCGCGU	21	-4.71	-4.94	TraesCS1A01G049600.2	ABC transporter G family member 5-like
						TraesCS1A01G276200.1	Glucanendo-1,3-beta-glucosidase 14
	novel_mir56	GGCCGCUGCACUCCUUGGCCGCUUGGGC	28	-2.1	-0.85		
	novel_mir57	UGGGCCUCACGGUCCAUAAU	20	-0.17	-1.12	TraesCS2D01G386500.1<br>TraesCS6B01G142400.1	Formin-like protein 3
						TraesCS5B01G183100.1<br>TraesCS5D01G190100.1	UDP-glycosyltransferase 90A1-like

Family name	miRNA name	Sequence	Length (nt)	Fold change [1]		Target gene	Annotation
				ST	SM		
	novel_mir58	CGAAGCGGCGCUCGGCCCCGG	21	-0.56	-1.81	TraesCS4B01G062900.1<br>TraesCS4D01G061900.1<br>TraesCS6A01G026800.1	polyol transporter 5-like<br><br>3-ketoacyl-CoA synthase 12-like
	novel_mir59	AUAUUUGCAGGUUUUAGGUCUAAGUGA	27	-0.27	1.54	TraesCS6B01G465600.1<br>TraesCS6B01G465600.2	CBL-interacting serine/threonine-protein kinase 7-like
	novel_mir60	GUGCUAGAUCCACGCCGCCGUUCAGUGUUG	30	-2.32	-0.65		
	novel_mir61	AACGGCCAGGAUUCGUCUGAU	21	-1.29	0.13	TraesCS4A01G480100.1	putative disease resistance RPP13-like protein 1
	novel_mir63	GUCGAUUAGUUUUAGCUUCAAGCCAUGCUA	30	-2.31	-2.24		
	novel_mir64	ACACUACGCGUGGAUGAGCA	20	-3.24	-0.91		
	novel_mir65	CAGAGCAGCGACAACUGCCCGCUUCCA		-1.6	-2.05		
	novel_mir66	UCUAGAGGAUGCAGUCUUUCCUCAAGG	27	-0.79	-2.2		
	novel_mir68	GACCGUACCCCAAACCGACA	20	0.34	-1.53		
	novel_mir69	CCCAGUAAACGGCGGCAGAGU	21	0	5.36	TraesCS7B01G138500.1	RNA pseudouridine synthase 3, mitochondrial
	novel_mir71	UGUGGCUUGUGGCUAAAACUAGUCUG	26	-1.64	-1.2		
	novel_mir75	CCAUCGUCGGCACGGCCGGUACCCACGCG	29	1.19	-4.75	TraesCS5A01G555600.1<br>TraesCS7D01G119200.1<br>TraesCS5D01G023700.1	hypothetical protein MTR_0021s0160

Family name	miRNA name	Sequence	Length (nt)	Fold change [1]		Target gene	Annotation
				ST	SM		
	novel_mir78	GGCGAGAGCGGGUCGCCGCGUGCCGGCC	28	-2.68	-2.04		

Table S2. Evolutionary conservation of known miRNA families in other plant species

"+" sign indicates the existence of miRNA in species, and "-" sign shows no homology in plant species

miRNA family	*Solanum lycopersicum*	*Solanum tuberosum*	*Arabidopsis thaliana*	*Pinus taeda*	*Physcomitrella patens*	*Medicago truncatula*	*Glycine max*	*Gossypium hirsutum*	*Populus trichocarpa*	*Brassica napus*	*Vitis vinifera*	*Citrus sinensis*	*Malus domestica*	*Cucumis melo*	*Zea may*	*Oryza sativa*	*Sorghum bicolor*	*Triticum aestivum*	*Aegilops tauschi*	*Triticum turgidum*	*Ricinus communis*	*Hordeum vulgare*	*Saccharum officinarum*	*Vigna unguiculata*	*Digitalis purpurea*	*Linum usitatissimum*	*Manihot esculenta*	*Prunus persica*	*Carica papaya*	*Cynara cardunculus*	*Theobroma cacao*	*Brachypodium distachyon*	*Salvia sclarea*	*Selaginella moellendorffii*	*Aquilegia caerulea*	*Festuca arundinacea*	*Hevea brasiliensis*	Total
MIR156	+	+	—	—	—	—	+	—	—	+	—	+	+	+	—	—	—	+	+	—	+	+	—	—	—	—	+	+	+	—	—	+	+	—	—	—	—	17
MIR159	+	—	+	+	—	+	+	—	+	+	+	+	+	+	+	+	+	+	—	—	+	+	+	—	—	+	+	+	+	—	—	+	—	+	+	+	+	27
MIR160	+	+	—	—	+	+	+	—	+	+	+	—	+	+	+	+	+	+	+	+	+	—	—	+	—	+	+	+	+	+	+	+	—	+	+	+	—	28
MIR164	+	+	+	—	—	+	+	+	+	+	+	+	+	+	+	+	+	+	+	—	+	—	—	+	—	+	+	+	+	+	+	+	+	—	—	+	—	28
MIR167	+	+	+	—	—	+	+	+	+	+	+	+	+	+	+	+	+	+	+	—	+	—	—	—	+	+	+	+	+	—	+	+	—	—	+	—	—	26
MIR169	+	+	+	—	—	+	+	+	+	+	+	+	+	+	+	+	+	+	+	—	+	+	—	+	—	+	+	+	+	+	+	+	+	—	+	+	—	30
MIR171	+	+	—	—	—	+	+	—	+	—	+	+	+	+	+	+	+	+	+	—	+	+	—	—	—	+	+	+	+	—	+	+	—	—	+	+	—	24
MIR395	—	+	—	—	—	—	—	—	—	—	—	+	+	+	—	+	+	+	+	—	—	—	—	—	—	—	+	+	+	—	—	+	—	—	—	—	—	12
MIR397	+	+	+	—	—	+	+	—	+	+	+	+	—	+	+	+	+	+	—	—	+	—	—	—	+	+	+	+	—	—	—	+	+	—	—	—	—	21
MIR398	+	+	+	+	—	+	+	+	+	—	+	+	+	+	+	+	+	+	+	—	+	—	—	—	—	+	+	+	+	+	+	+	+	—	+	—	+	28
MIR399	+	+	+	—	—	+	+	+	+	+	+	+	+	+	+	+	+	+	+	—	+	+	—	+	—	+	+	+	—	+	+	+	—	—	+	—	—	27
MIR408	+	+	+	+	+	+	+	—	+	—	+	+	+	+	+	+	+	+	+	—	+	—	+	+	+	+	+	—	+	+	—	+	+	+	+	—	+	30
MIR444	—	—	—	—	—	—	—	—	—	—	—	—	—	—	+	+	—	+	—	—	—	+	—	—	—	—	—	—	—	—	—	+	—	—	—	—	—	5
MIR1119	—	—	—	—	—	—	—	—	—	—	—	—	—	—	—	—	—	+	—	—	—	—	—	—	—	—	—	—	—	—	—	—	—	—	—	+	—	2
MIR1120	—	—	—	—	—	—	—	—	—	—	—	—	—	—	—	—	—	+	—	—	—	+	—	—	—	—	—	—	—	—	—	—	—	—	—	—	—	2
MIR1122	—	—	—	—	—	—	—	—	—	—	—	—	—	—	—	—	—	+	—	—	—	—	—	—	—	—	—	—	—	—	—	+	—	—	—	+	—	3
MIR1139	—	—	—	—	—	—	—	—	—	—	—	—	—	—	—	—	—	+	—	—	—	—	—	—	—	—	—	—	—	—	—	+	—	—	—	—	—	2
MIR5050	—	—	—	—	—	—	—	—	—	—	—	—	—	—	—	—	—	+	—	—	—	+	—	—	—	—	—	—	—	—	—	—	—	—	—	—	—	2
MIR5062	—	—	—	—	—	—	—	—	—	—	—	—	—	—	—	—	—	+	+	—	—	—	—	—	—	—	—	—	—	—	—	+	—	—	—	—	—	3
MIR5067	—	—	—	—	—	—	—	—	—	—	—	—	—	—	—	—	—	—	—	—	—	—	—	—	—	—	—	—	—	—	—	+	—	—	—	—	—	1

**miRNA family**	***Solanum lycopersicum***	***Solanum tuberosum***	***Arabidopsis thaliana***	***Pinus taeda***	***Physcomitrella patens***	***Medicago truncatula***	***Glycine max***	***Gossypium hirsutum***	***Populus trichocarpa***	***Brassica napus***	***Vitis vinifera***	***Citrus sinensis***	***Malus domestica***	***Cucumis melo***	***Zea may***	***Oryza sativa***	***Sorghum bicolor***	***Triticum aestivum***	***Aegilops tauschi***	***Triticum turgidum***	***Ricinus communis***	***Hordeum vulgare***	***Saccharum officinarum***	***Vigna unguiculata***	***Digitalis purpurea***	***Linum usitatissimum***	***Manihot esculenta***	***Prunus persica***	***Carica papaya***	***Cynara cardunculus***	***Theobroma cacao***	***Brachypodium distachyon***	***Salvia sclarea***	***Selaginella moellendorffii***	***Aquilegia caerulea***	***Festuca arundinacea***	***Hevea brasiliensis***	**Total**
MIR5084	—	—	—	—	—	—	—	—	—	—	—	—	—	—	—	—	—	+	+	—	—	—	—	—	—	—	—	—	—	—	—	—	—	—	—	—	—	2
MIR5200	—	—	—	—	—	—	—	—	—	—	—	—	—	—	—	—	—	+	+	—	—	—	—	—	—	—	—	—	—	—	—	+	—	—	—	—	—	3
MIR6201	—	—	—	—	—	—	—	—	—	—	—	—	—	—	—	—	—	+	+	—	—	+	—	—	—	—	—	—	—	—	—	—	—	—	—	—	—	3
MIR7757	—	—	—	—	—	—	—	—	—	—	—	—	—	—	—	—	—	+	+	—	—	—	—	—	—	—	—	—	—	—	—	—	—	—	—	—	—	2
MIR818	—	—	+	—	—	—	—	—	—	—	—	—	—	—	—	+	—	—	—	—	—	—	—	—	—	—	—	—	—	—	—	—	—	—	—	—	—	2
MIR9654	—	—	—	—	—	—	—	—	—	—	—	—	—	—	—	—	—	+	—	—	—	—	—	—	—	—	—	—	—	—	—	—	—	—	—	—	—	1
MIR9657	—	—	—	—	—	—	—	—	—	—	—	—	—	—	—	—	—	+	—	—	—	—	—	—	—	—	—	—	—	—	—	—	—	—	—	—	—	1
MIR9662	—	—	—	—	—	—	—	—	—	—	—	—	—	—	—	—	—	+	—	—	—	—	—	—	—	—	—	—	—	—	—	—	—	—	—	—	—	1
MIR9666	—	—	—	—	—	—	—	—	—	—	—	—	—	—	—	—	—	+	—	—	—	—	—	—	—	—	—	—	—	—	—	—	—	—	—	—	—	1
MIR9672	—	—	—	—	—	—	—	—	—	—	—	—	—	—	—	—	—	+	+	—	—	—	—	—	—	—	—	—	—	—	—	—	—	—	—	—	—	2
MIR9674	—	—	—	—	—	—	—	—	—	—	—	—	—	—	—	—	—	+	+	—	—	—	—	—	—	—	—	—	—	—	—	—	—	—	—	—	—	2
MIR9677	—	—	—	—	—	—	—	—	—	—	—	—	—	—	—	—	—	+	+	—	—	—	—	—	—	—	—	—	—	—	—	—	—	—	—	—	—	2
MIR9772	—	—	—	—	—	—	—	—	—	—	—	—	—	—	—	—	—	+	+	—	—	—	—	—	—	—	—	—	—	—	—	—	—	—	—	—	—	2
MIR9776	—	—	—	—	—	—	—	—	—	—	—	—	—	—	—	—	—	+	+	—	—	—	—	—	—	—	—	—	—	—	—	—	—	—	—	—	—	2
MIR9783	—	—	—	—	—	—	—	—	—	—	—	—	—	—	—	—	—	+	+	—	—	—	—	—	—	—	—	—	—	—	—	—	—	—	—	—	—	2

Table S3. Description, expression and putative target genes of known miRNAs in roots whose expression were unchanged in Suntop (ST) but up-regulated in Sunmate (SM), or down-regulated in ST but up-regulated or unchanged in SM treated with 100 mM NaCl for 1 day (100 mM NaCl vs control).

Family name	miRNA name	Sequence	Length (nt)	Fold change [1]		Target gene	Annotation
				ST	SM		
MIR156	Tae-miR156	UGACAGAAGAGAGUGAGCACA	21	-1.28	-0.73	TraesCS2A01G350100.1	Nitrate transporter 1.2
						TraesCS2B01G368600.1	
						TraesCS2B01G368600.2	
						TraesCS2D01G348500.1	protein NRT1/ PTR FAMILY 4.6-like isoform X1
						TraesCS2D01G348500.2	
						TraesCS2D01G348500.3	
						TraesCS6A01G110100.2	squamosa promoter-binding-like protein 3
						TraesCS6B01G138400.1	
						TraesCS6D01G098500.1	
						TraesCS6D01G145200.1	squamosa promoter-binding-like protein 4
MIR159	tae-miR159a,	UUUGGAUUGAAGGGAGCUCUG	21	-0.39	1.92	TraesCS3A01G131700.1	protein SPEAR2
	tae-miR159b					TraesCS3A01G131700.3	
						TraesCS3D01G147900.1	
						TraesCS3D01G147900.2	
						TraesCS3A01G336500.1	transcription factor GAMYB
						TraesCS3D01G329400.1	
						TraesCS3D01G329400.2	

Family name	miRNA name	Sequence	Length (nt)	Fold change [1]		Target gene	Annotation
				ST	SM		
						TraesCS3D01G329400.3	
						TraesCS3B01G367500.1	R2R3-MYB protein
						TraesCS3B01G367500.2	
MIR160	tae-miR160	UGCCUGGCUCCCUGUAUGCCA	21	-1.42	1.58	TraesCS2A01G380300.1	auxin response factor 8-like
						TraesCS2B01G397300.1	
						TraesCS4B01G346200.1	protein FIZZY-RELATED 2-like
						TraesCS4B01G346200.2	
						TraesCS4D01G341200.1	
MIR171	tae-miR171a	UGAUUGAGCCGUGCCAAUAUC	21	-2.46	1.46	TraesCS1A01G209400.1	Scarecrow-like protein 6
						TraesCS1B01G223400.1	
						TraesCS1D01G212800.1	
	tae-miR171b	UUGAGCCGUGCCAAUAUCACG	21	-1.03	-0.89	TraesCS6A01G247200.1	Scarecrow protein 6
						TraesCS6B01G277400.1	
						TraesCS6D01G229400.1	
						TraesCS2A01G352600.1	6,7-dimethyl-8-ribityllumazine synthase, chloroplastic
						TraesCS2B01G371700.1	
						TraesCS2D01G351600.1	
MIR395	tae-miR395b	UGAAGUGUUUGGGGGAACUC	20	-1.29	-0.16	TraesCS5A01G382900.1	ATP sulfurylase
	tae-miR395a	GUGAAGUGUUUGGGGGAACUC	21	-0.45	3.49	TraesCS5B01G387300.1	ATP sulfurylase 4,
						TraesCS5D01G392300.1	chloroplastic-like

Family name	miRNA name	Sequence	Length (nt)	Fold change [1]		Target gene	Annotation
				ST	SM		
MIR818	tae-miR1130b-3p	UCUUAUAUUAUGGGACGGAGG	21	-0.22	1.09	TraesCS2D01G248200.6	Inactive leucine-rich repeat receptor-like protein kinase
						TraesCS2A01G366400.1	hypothetical protein OsI_25001
						TraesCS2A01G366400.2	hypothetical protein F775_04208
MIR5048	tae-miR5048-5p	UUUGCAGGUUUUAGGUCUAAGU	22	-0.72	1.24	TraesCS3A01G521100.1	uncharacterized protein LOC109783670
						TraesCS3A01G521100.2	
						TraesCS4B01G313900.2	
						TraesCS4B01G313900.3	
						TraesCS1B01G119300.3	starch synthase III
						TraesCS7D01G487800.1	GDSL esterase/lipase
MIR5062	tae-miR5062-5p	UGAACCUUAGGGAACAGCCGCAU	23	-0.77	1.34		
MIR5067	tae-miR5175-5p	UUCCAAAUUACUCGUCGUGGU	21	-0.32	1.4	TraesCS2A01G485700.2	receptor-like serine/threonine-protein kinase SD1-8
MIR5200	tae-miR5200	UGUAGAUACUCCCUAAGGCUU	21	-0.09	1.20	TraesCS5D01G072900.1	hypothetical protein F775_12042
MIR7757	tae-miR7757-5p	AUAAAACCUUCAGCUAUCCAUC	22	-1.10	1.78	TraesCS2A01G023400.1	putative disease resistance protein At1g50180
						TraesCS2B01G033500.1	
						TraesCS2D01G024500.1	
						TraesCS2D01G024500.2	

Family name	miRNA name	Sequence	Length (nt)	Fold change [1]		Target gene	Annotation
				ST	SM		
MIR9652	tae-miR9652-5p	CCUGUUUGUCAUUAAGUUUCUU	22	-0.54	1.41	TraesCS3B01G287600.2	protein argonaute MEL1
						TraesCS5B01G451400.1	
						TraesCS5B01G452500.1	
MIR9653	tae-miR9653b	UGGCCAAGGUCUCUUGAGGCU	21	-1.07	1.80	TraesCS1A01G228200.1	probable protein phosphatase 2C 48
						TraesCS1A01G228200.2	
MIR9654	tae-miR9654b-3p	UUCCGAAAGGCUUGAAGCGAAU	22	-0.9	2.18	-	-
MIR9656	tae-miR9656-3p	CUUCGAGACUCUGAACAGCGG	21	-0.17	1.72	-	-
MIR9657	tae-miR9657a-3p	UGUGCUUCCUCGUCGAACGGU	21	-0.31	1.27	TraesCS3B01G280400.1	WD-40 repeat-containing protein MSI4 isoforms
						TraesCS3D01G251300.1	
						TraesCS3A01G250900.2	
						TraesCS7B01G144300.1	MYB-related protein
						TraesCS7B01G006500.1	putative serpin-Z5
						TraesCS7D01G103400.1	
						TraesCS3A01G250900.3	FVE [*Triticum aestivum*]
						TraesCS3A01G250900.1	
	tae-miR9657c-3p, tae-miR9657b-3p	CGUGCUUCCUCGUCGAACGGU	21	-0.38	1.16	TraesCS3B01G280400.1	WD-40 repeat-containing protein MSI4 isoforms
						TraesCS3D01G251300.1	
						TraesCS3A01G250900.2	
						TraesCS7B01G006500.1	putative serpin-Z5
						TraesCS7D01G103400.1	

Family name	miRNA name	Sequence	Length (nt)	Fold change [1]		Target gene	Annotation
				ST	SM		
						TraesCS7B01G144300.1	MYB-related protein
						TraesCS3A01G250900.1	FVE [*Triticum aestivum*]
						TraesCS3A01G250900.3	
	tae-miR9662b-3p	UGAACAUCCCAGAGCCACCGG	21	-0.09	1.45	TraesCS6B01G021800.1	uncharacterized protein LOC109765037
						TraesCS6B01G043100.1	
						TraesCS6D01G035100.1	cytosolic sulfotransferase 5-like
MIR9664	tae-miR9664-3p	UUGCAGUCCUCGAUGUCGUAG	21	-0.64	1.04	TraesCS3A01G247700.1	cyclin dependent protein kinase
						TraesCS7D01G096000.1	disease resistance protein
						TraesCS5D01G405600.1	
						TraesCS2D01G541000.1	
MIR9666	tae-miR9666a-3p	CGGUAGGGCUGUAUGAUGGCGA	22	-1.71	-0.69	TraesCS5B01G438300.1	BTB/POZ and MATH domain-containing protein 3
	tae-miR9666c-5p,	GCCAUCAUACGUCCAACCGUG	21	-2.43	-0.69	TraesCS2B01G562600.1	exocyst complex
	tae-miR9666b-5p						component EXO70A1-like
MIR9668	tae-miR9668-5p	CCAAUGACAAGUAUUUUCGGA	21	-0.28	1.75	TraesCS6B01G070200.1	disease resistance protein RGA2-like
MIR9669	tae-miR9669-5p	UACUGUGGGCACUUAUUUGAC	21	-0.72	2.13	-	-
MIR9672	tae-miR9672b	UACCACGACUGUCAUUAAGCA	21	-1.12	-0.57	TraesCS7D01G383200.1	replication factor C subunit 1

Family name	miRNA name	Sequence	Length (nt)	Fold change [1]		Target gene	Annotation
				ST	SM		
MIR9674	tae-miR9674b-5p	AUAGCAUCAUCCAUCCUACCC	21	-0.08	1.48		
						TraesCS2A01G531000.1<br>TraesCS6A01G014800.1<br>TraesCS6B01G021600.1<br>TraesCS6D01G017700.1	protein Rf1, mitochondrial-like
MIR9675	tae-miR9675-3p	UUUAUGAUCACUCUCGUUUUG	21	-0.21	2.48	TraesCS3A01G074300.1	premnaspirodiene oxygenase-like
MIR9772	tae-miR9772	UGAGAUGAGAUUACCCCAUAC	21	-0.58	1.7	TraesCS6A01G063700.1<br>TraesCS6B01G085300.1<br>TraesCS6B01G085300.2	F-box protein At5g03970-like
						TraesCS7D01G005400.1<br>TraesCS6A01G058900.1	F-box/kelch-repeat protein At3g61590-like
MIR9773	tae-miR9773	UUUGUUUUUAUGUUAUUUUGUGAA	24	-0.39	2.63	-	-
MIR9775	tae-miR9775	UGUGCGCAAUAAGAUUUUGCUA	22	0.46	1.97	TraesCS7D01G005400.1	F-box/kelch-repeat protein SKIP11

[1] Fold change (NaCl *vs* control) is $\log_2 N$, $\log_2 N \geqslant 1$ are up-regulated, between $0 < |\log_2 N| < 1$ are unchanged and $\log_2 N \leqslant -1$ are down-regulated, *P*-values 0.01 and Q-value ⩽ 0.001.

Table S4. Description, expression and putative target genes of known miRNAs in roots whose expression were unchanged in Suntop (ST) but down-regulated in Sunmate (SM), or up-regulated in ST but down-regulated or unchanged in SM treated with 100 mM NaCl for 1 day.

miRNA family	miRNA name	Sequence	Length (nt)	Fold change [1]		Target gene	Annotation
				ST	SM		
MIR319	tae-miR319	UUGGACUGAAGGGAGCUCCCU	21	-0.21	-2.97	TraesCS3D01G146900.1<br>TraesCS3A01G140100.1	transcription factor PCF5-like

miRNA family	miRNA name	Sequence	Length (nt)	Fold change [1]		Target gene	Annotation
				ST	SM		
MIR397	tae-miR397-5p	UCACCGGCGCUGCACACAAUG	21	1.76	-0.69	TraesCS7A01G047900.1	probable protein phosphatase 2C 37
MIR1117	tae-miR1117	UAGUACCGGUUCGUGGCACGAACC	24	-0.32	-1.47		
MIR1125	tae-miR1125	AACCAACGAGACCAACUGCGGCGG	24	0.28	-2.41		
	tae-miR1137a	UAGUACAAAGUUGAGUCAUC	20	0.25	-1.53	TraesCS6A01G107500.1<br>TraesCS6B01G136100.1<br>TraesCS6B01G136200.1	unnamed protein product
MIR9652	tae-miR9652-3p	AAGCUUAAUGAGAACAUGUG	20	1.24	0		
MIR9658	tae-miR9658-3p	AUCGUUCUGGGUGAAUAGGCC	21	-0.25	-2.03		
MIR9672	tae-miR9672a-3p	CCACGACUGUCAUUAAGCAUC	21	0.65	-1.02		
MIR9778	tae-miR9778	UGCAUCAUCUCGAACUCGUCG	21	-0.62	-2	TraesCS1A01G021700.1<br>TraesCS1A01G021800.1<br>TraesCS1A01G021800.2<br>TraesCS1B01G025500.1<br>TraesCS1B01G027000.1<br>TraesCS1B01G027500.1<br>TraesCS1B01G027500.2	Disease resistance protein RPM1

[1] Fold change (NaCl *vs* control) is log_2N, $log_2N \geqslant 1$ are up-regulated, between $0 < |log_2N| < 1$ are unchanged and $log_2N \leqslant -1$ are down-regulated, *P*-values 0.01 and Q-value $\leqslant$ 0.001.

Table S5. Description, expression and putative target genes of novel miRNAs in roots whose expression were unchanged in Suntop (ST) but up-regulated in Sunmate (SM), or down-regulated in ST but up-regulated (-) or unchanged in SM treated with 100 mM NaCl for 1 day

miRNA name	Sequence	Length (nt)	Fold change [1]		Target gene	Annotation
			ST	SM		
novel_mir8	UCCCGGCCCCGAACCUGUCGGCU		-0.37	1.17	TraesCS6A01G114800.1<br>TraesCS6B01G142400.1	premnaspirodiene oxygenase-like
novel_mir14	AGAGAUCCCGCGCGCUACUCCGUCGA	26	-1.53	-0.49		
novel_mir16	CUUGAAGACUUUGGCCACGUCCAU	24	-1.69	-0.68		
novel_mir17	ACCUUUGAGACUUUGGCCAUGGCCA	25	-1.83	-0.94		

miRNA name	Sequence	Length (nt)	Fold change [1]		Target gene	Annotation
			ST	SM		
novel_mir18	UUGAAGACUUUGGCCAUGUCCAUGGUG	27	-1.34	-0.99		
novel_mir24	AUGGGCUACGUGUGUUAAAC	20	-2.3	-0.69		
			2.34			
novel_mir28	ACUGGUUGGAUCAUGCUUCUGUUUAUGA	26	-0.87	1.38	TraesCS1D01G090600.1	putative disease resistance RPP13-like protein 3
					TraesCS6B01G387200.1	Disease resistance protein RPM1
novel_mir32	UGUUUGGUGCGGACUCUGGACCU	23	-1.23	-0.96		
novel_mir33	AAGAACAUCUAAGGGGCUGAGUUG	24	-1.56	-0.23		
novel_mir34	AAAAUGAUGUGUUGUGGAAAGCA	23	-1.91	1.87		
novel_mir36	UUGGCGAUAGCGAAUGCAGUUCUC	24	-2.31	-0.16		
novel_mir40	GUGCACCCGUGUUCACCUUUGUAUUU	26	-0.28	1.93		
novel_mir45	UGAUUAACGGCCAGGAUUUCCCUG	24	-0.13	1.12		
novel_mir48	AUGGAUCUGACGGCUGUAGAGGA	23	-0.43	1.16		
novel_mir49	GUGAUUAACGGCCAGGAUUUCCCUG	25	-2.18	1.67		
novel_mir56	GGCCGCUGCACUCCUUGGCCGCUUGGGC	28	-2.19	-0.85		
novel_mir59	AUAUUUGCAGGUUUUAGGUCUAAGUGA	27	-0.27	1.54	TraesCS6B01G465600.1	CBL-interacting serine/threonine-
					TraesCS6B01G465600.2	protein kinase 7-like
novel_mir60	GUGCUAGAUCCACGCCGCCGUUCAGUGUUG	30	-2.32	-0.65		
novel_mir61	AACGGCCAGGAUUCGUCUGAU	21	-1.29	0.13	TraesCS4A01G480100.1	putative disease resistance RPP13-like protein 1

miRNA name	Sequence	Length (nt)	Fold change [1]		Target gene	Annotation
			ST	SM		
novel_mir64	ACACUACGCGUGGAUGAGCA	20	-3.24	-0.91		

[1] Fold change (NaCl *vs* control) is log_2N, $log_2N \geqslant 1$ are up-regulated, between $0 < |log_2N| < 1$ are unchanged and $log_2N \leqslant -1$ are down-regulated, *P*-values 0.01 and Q-value $\leqslant$ 0.001.

Table S6. Description, expression and putative target genes of novel miRNAs in roots whose expression were unchanged in Suntop (ST) but down-regulated (-) in Sunmate (SM), or up-regulated in ST but down-regulated or unchanged in SM treated with 100 mM NaCl for 1 day

miRNA name	Sequence	Length (nt)	Fold change [1]		Target gene	Annotation
			ST	SM		
novel_mir9	UUUGAAGACUAGUUUAUUAUAU	22	-0.36	-2		
novel_mir11	ACGCAUCAUUCAAAUUUCUGCCCUAUCA	28	-0.23	-1.42		
novel_mir19	UCCCACAGUGCAAAUUUAAAUUUUAUUAGA	30	-0.45	-1.01		
novel_mir23	AGCACCGUUGGCAUCAUCGUCGGCGUUGUC	30	-0.57	-1.16		
novel_mir25	UUGGGUUCGAGCCCCAAGGUGGG	23	1.69	0.98		
novel_mir29	AGUUCGAGUCGGAGGCCACGGUGCUGGG	28	-0.33	-1.47		
novel_mir30	UUGUUUGGCCAUCAAGAACAAUAGUAG	27	-0.36	-1.32		
novel_mir31	AUCGAGAAAUUGGAGCUCGGUGCAGGCAUA	30	-0.62	-1.39		
novel_mir37	AAGUUGCGUAGUGGAUCGCUUGGGGCCUA	29	-0.78	-1.18		
novel_mir46	GCGGCGACGGGGGCGGCUU	19	3.21	-1.39		
novel_mir53	CCGGAUGUGUUGUCUUCCUCGACCAUGGUG	30	-0.01	-1.3		
novel_mir57	UGGGCCUCACGGUCCAUAAU	20	-0.17	-1.12	TraesCS2D01G386500.1	Formin-like protein 3
					TraesCS6B01G142400.1	
					TraesCS5B01G183100.1	UDP-glycosyltransferase 90A1-like
					TraesCS5D01G190100.1	
novel_mir58	CGAAGCGGCGCUCGGCCCCGG	21	-0.56	-1.81	TraesCS4B01G062900.1	polyol transporter 5-like
					TraesCS4D01G061900.1	
novel_mir66	UCUAGAGGAUGCAGUCUUUCCUCAAGG	27	-0.79	-2.2		
novel_mir68	GACCGUACCCCAAACCGACA	20	0.34	-1.53		

miRNA name	Sequence	Length (nt)	Fold change [1]		Target gene	Annotation
			ST	SM		
novel_mir75	CCAUCGUCGGCACGGCCGGUACCCACGCG	29	1.19	-4.75	TraesCS5A01G555600.1<br>TraesCS7D01G119200.1<br>TraesCS5D01G023700.1	hypothetical protein MTR_0021s0160

[1] Fold change (NaCl *vs* control) is $\log_2 N$, $\log_2 N \geqslant 1$ are up-regulated, between $0 < |\log_2 N| < 1$ are unchanged and $\log_2 N \leqslant -1$ are down-regulated, *P*-values 0.01 and Q-value $\leqslant$ 0.001.

Table S7. List of gene specific primers used in qRT PCR analysis

miRNA	Target gene	Primers	Amplicon size
tae-mir160	TraesCS2B01G397300.1	**F-**GGATCTCCACCTCAGCAACC<br>**R-**CGTGATGTTGTCGTCTTCGC	154
tae-miR319	TraesCS3A01G140100.1	**F-**GAAGGACCGGCACAGTAAG<br>**R-**TCCTTGGCGTTCTTGATGAG	159
tae-miR397-5p	TraesCS7A01G047900.1	**F-**GAACAGATGCACACCATCTTG<br>**R-**ATGGGCCATCCCAATTCC	149
tae-mir9653b	TraesCS1A01G228200.1	**F-**GTCAAGGACTACGGCGTGAT<br>**R-**AATCTGTCGCTTACCCCGTC	104
tae-miR9657a-3p	TraesCS7B01G144300.1	**F-**CGATCATTGTTTGCCGGAAG<br>**R-**ACCCTTGTTGGTCCTCATAC	161
tae-miR9772	TraesCS6A01G058900.1	**F-**GGCTTTCTTGCGGACATTTG<br>**R-**CCACCACCAATGCTTGTTTC	141
tae-miR9778	TraesCS1D01G021300.1	**F-**GTGTGGCAAAGCAGATGAAG<br>**R-**ACACCGCAATCACCCTAAG	168
Novel_mir8	TraesCS6A01G142400.1	**F-**CATCGACACAAGGGCTGGA<br>**R-**TCTTGGTGGTGCCTGTTTCTT	**75**
	Actin	**F-**CACTGGAATGGTCAAGGCTG<br>**R-**CTCCATGTCATCCCAGTTG	

Table S8. List of miRNA primers used in qRT PCR analysis

miRNA	Primer sequence	Length
Tae_miR156	UGACAGAAGAGAGUGAGCACA	21
Tae_miR171a	UGAUUGAGCCGUGCCAAUAUC	21
Tae_miR319	UUGGACUGAAGGGAGCUCCCU	21
Tae_miR395b	UGAAGUGUUUGGGGGAACUC	20
Tae_miR397-5p	UCACCGGCGCUGCACACAAUG	21
Novel_miR59	AUAUUUGCAGGUUUUAGGUCUAAGUGA	27
Novel_miR 75	CCAUCGUCGGCACGGCCGGUACCCACGCG	29

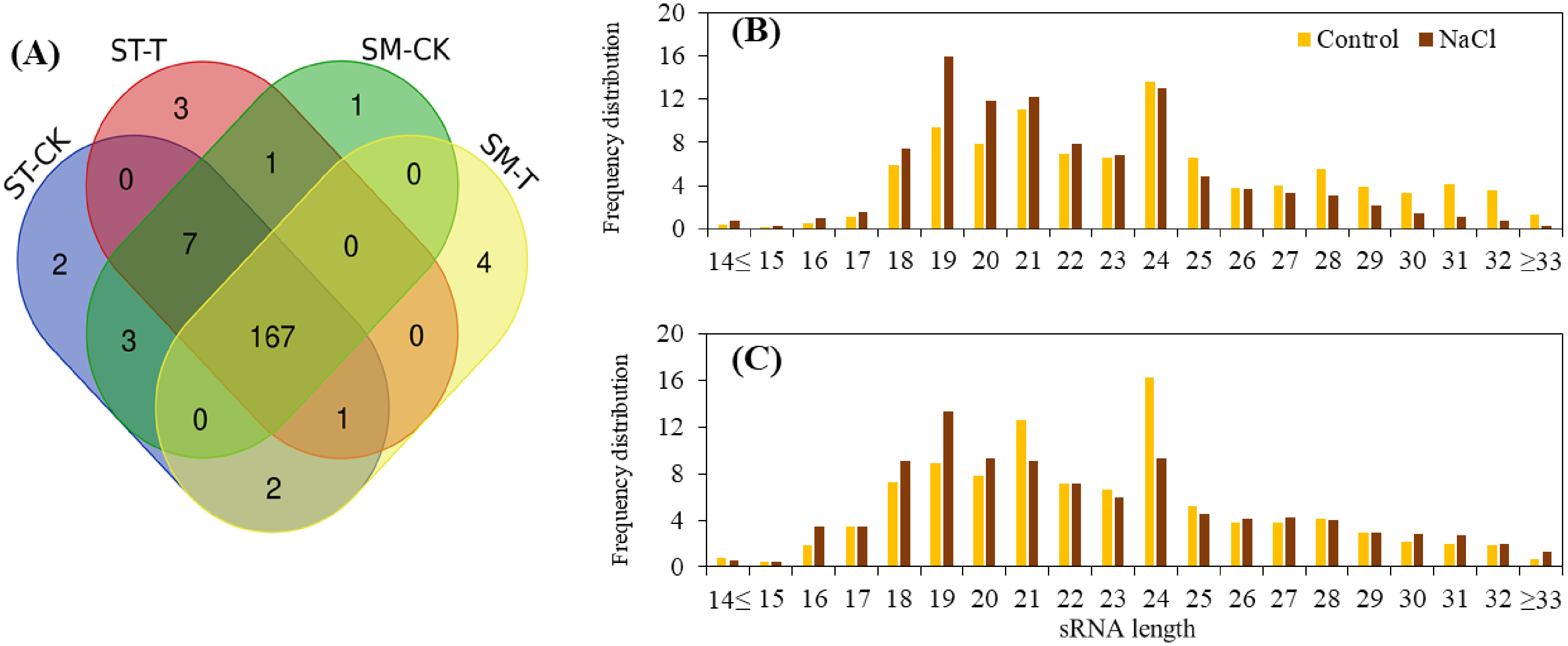

Figure S1. Number of miRNAs in two wheat cultivars under NaCl stress (T) and control conditions (CK) were counted in root **(A).** Length base distribution of total identified miRNAs in roots of two wheat cultivars **(B**) Suntop (ST)，**(C)** Sunmate (SM).

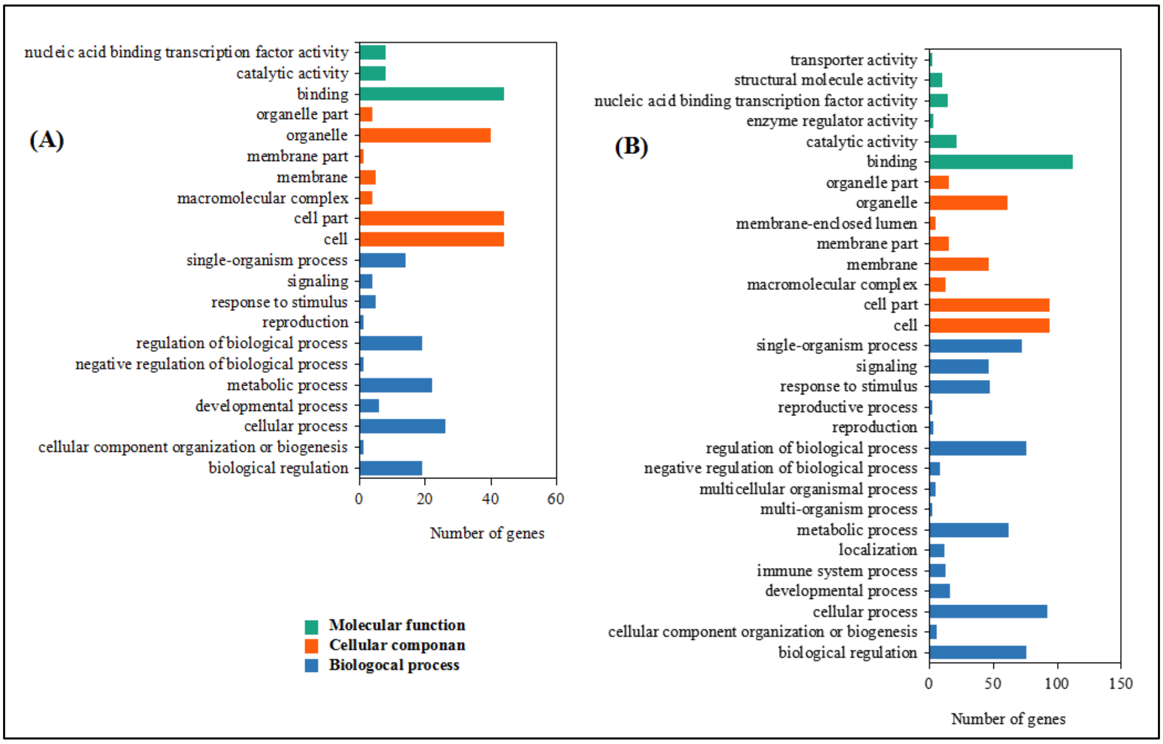

Figure S2. Go analysis of identified target genes in cultivar Suntop **(A)** and Sunmate **(B).** Target genes were classified into three categories: biological processes, cellular component, and molecular function. Values in the Y axis are the number of target genes in different functional categories
